# Supplementary material for: Genomic analyses of 10,376 individuals in the Westlake BioBank for Chinese (WBBC) pilot project
Source: Nat Commun. 2022 May 26;13:2939. doi: 10.1038/s41467-022-30526-x (PMC9135724; doi:10.1038/s41467-022-30526-x)
Supplement: Supplementary file 1 — Supplementary Information [file 41467_2022_30526_MOESM1_ESM.pdf]

Supplementary Information

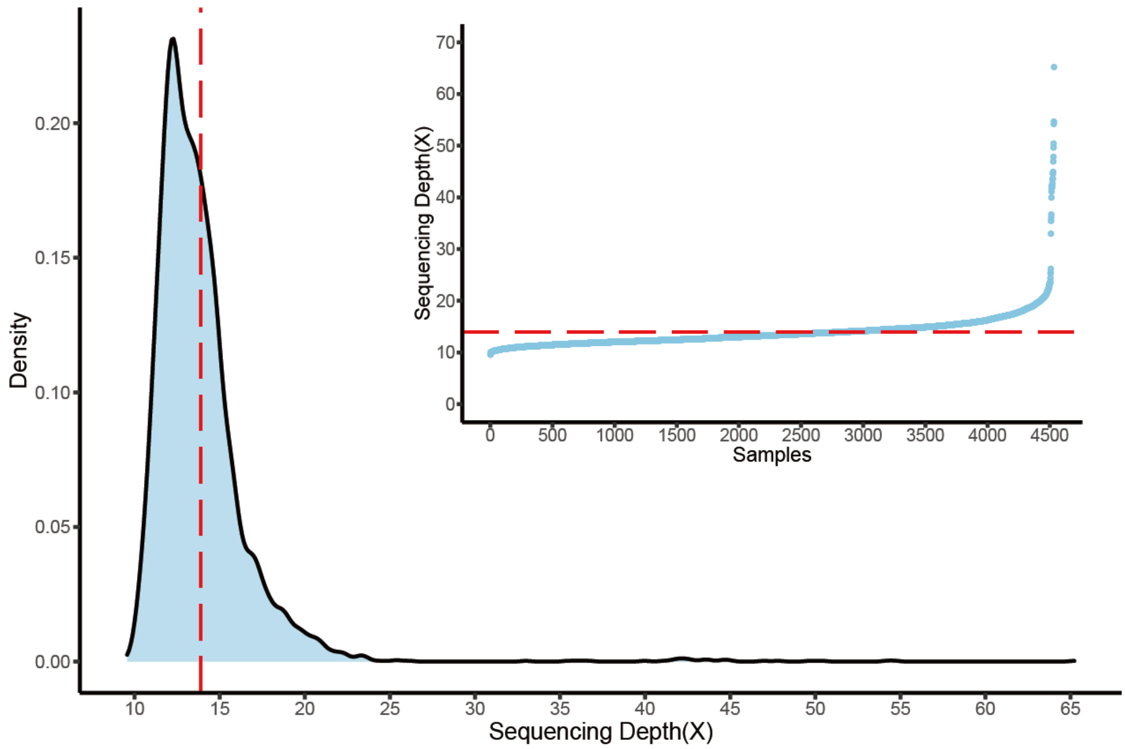

Supplementary Figure 1. The basic statistics of samples and variants in the WBBC-cohort.

The sequencing depth of all 4,480 samples. The central red dot lines are the median. The inner chart represents the sequencing depth by sorted samples. The area plot indicates the density of sequencing depth. Source data are provided as a Source Data file.

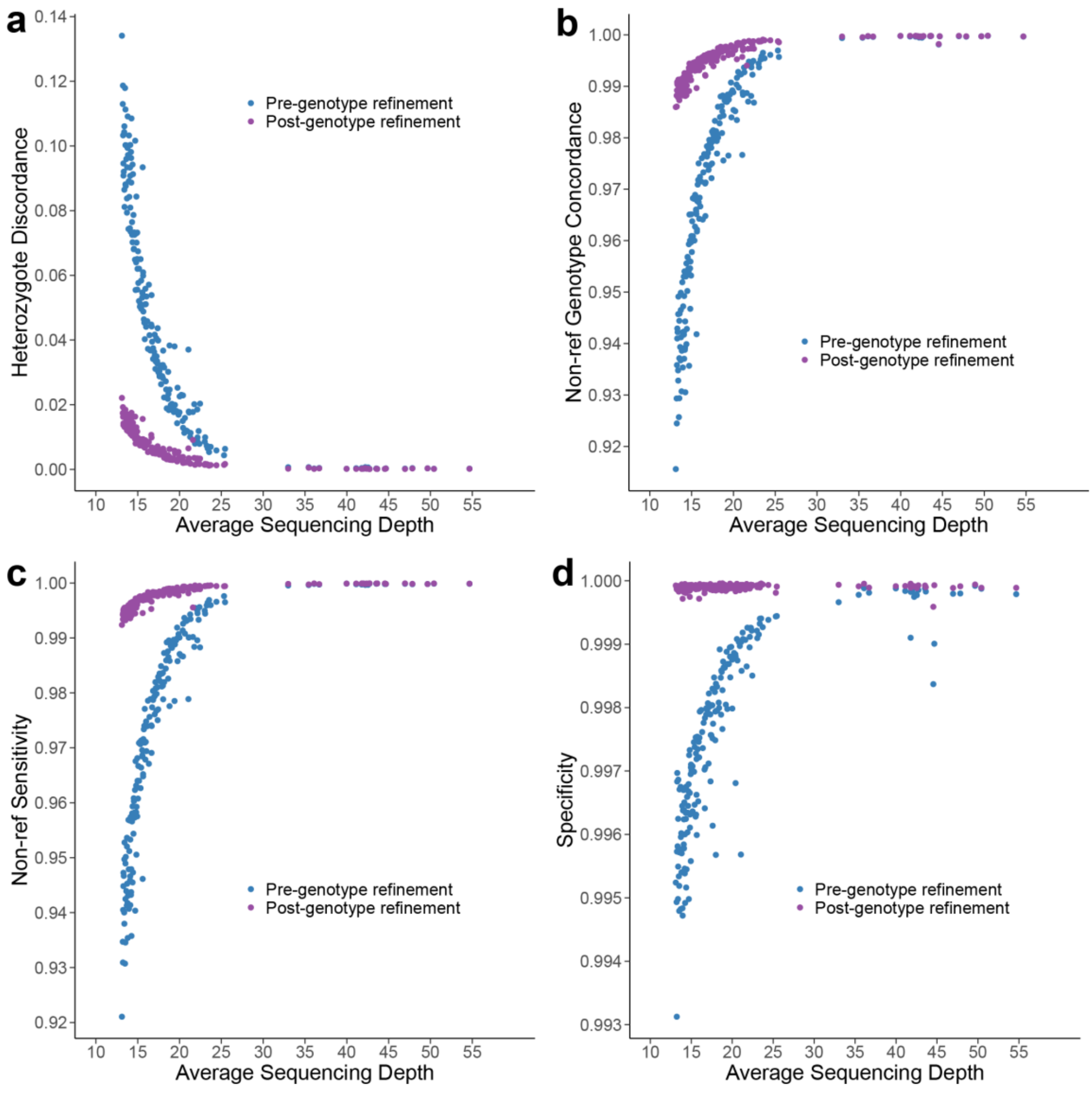

Supplementary Figure 2. A comparison of the genotype concordance between WGS and SNP array data.

a Heterozygote discordance rate. **b** Non-reference genotype concordance. **c** Non-reference sensitivity. **d** Specificity. The LD-based genotype refinement was conducted by BEAGLE software version 5.1. Source data are provided as a Source Data file.

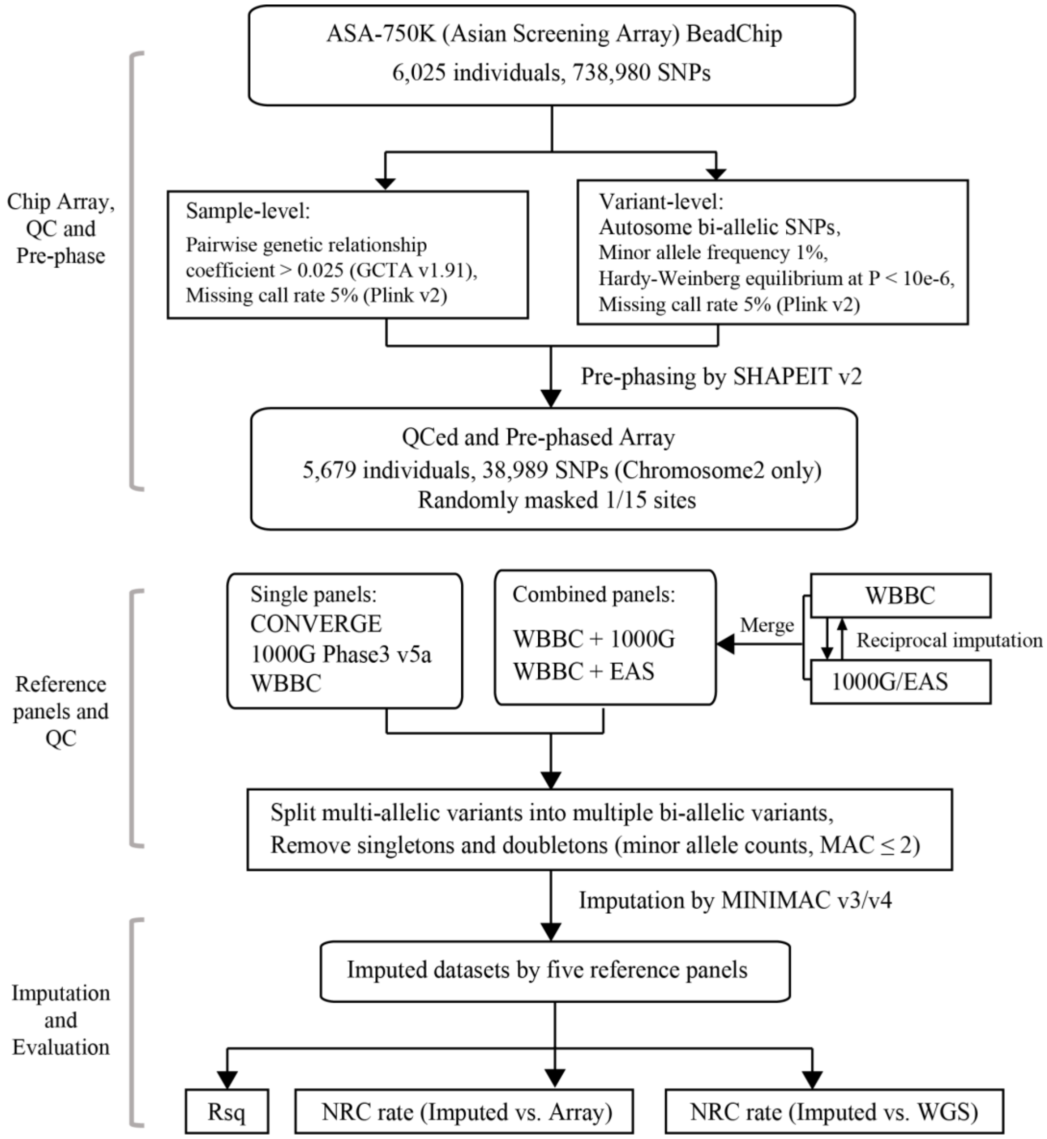

Supplementary Figure 3. Imputation evaluation workflow.

Rsq: R-square value calculated by Minimac4. NRC rate: non-reference allele genotype concordance rate. 1000G included 3,284,591 variants and 5,008 haplotypes; CONVERGE included 1,115,342 variants and 23,340 haplotypes. The WBBC included 2,089,508 variants and 8,610 haplotypes. The WBBC+1000G combined panel consisted of 13,618 haplotypes with 4,450,989 variants. The WBBC+EAS combined panel consisted of 9,618 haplotypes with 2,411,382 variants.

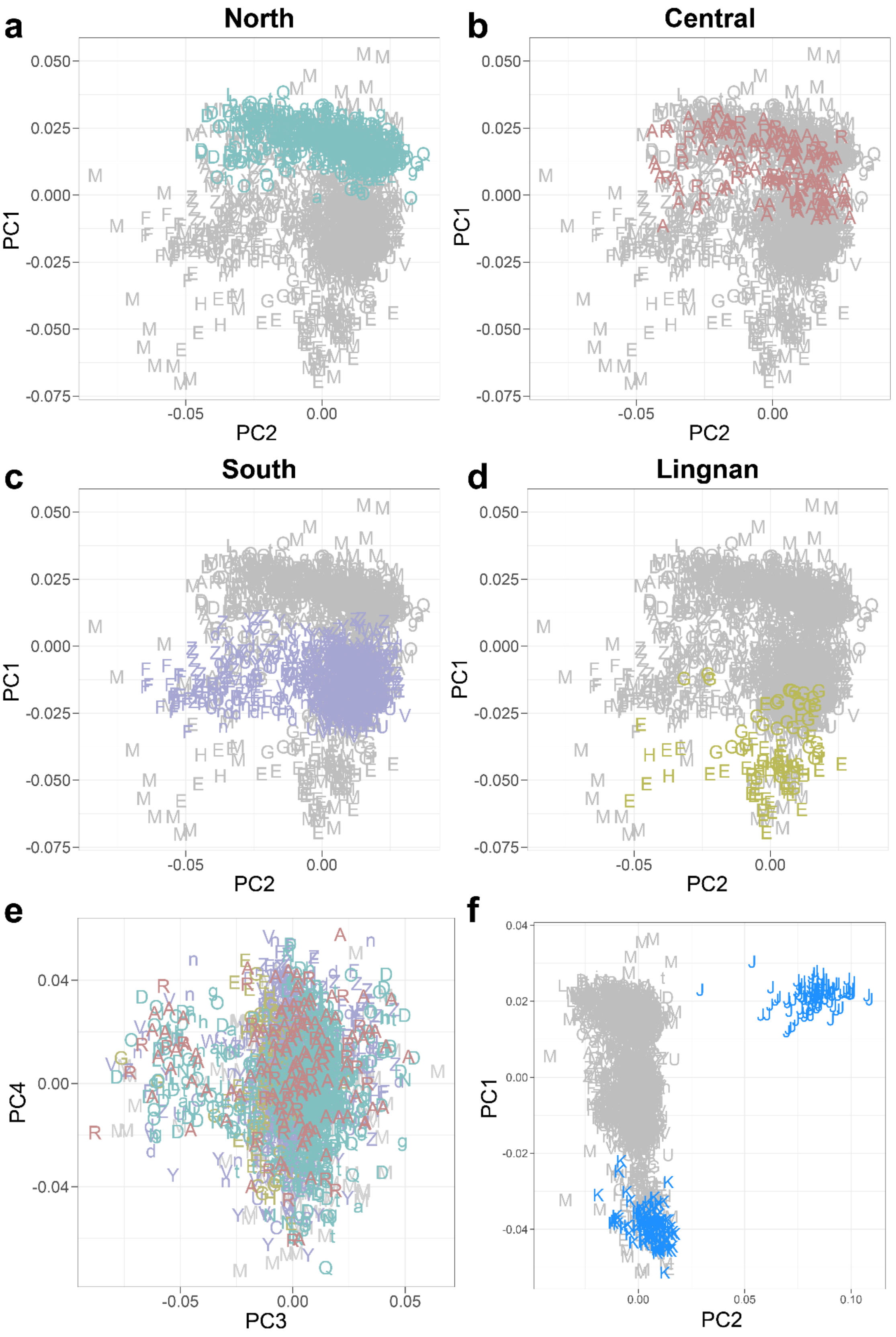
a
North
b
Central
c
South
d
Lingnan
e
f
PC1
PC2
PC3
PC4

Supplementary Figure 4. Principal Component Analysis (PCA).

The administrative divisions are shown by the distinct letters. Minority people are marked with "M". The Han Chinese populations can be classified into four subgroups: **a** North Han (Gansu-g, Hebei-h, Heilongjiang-r, Henan-O, Inner Mongolia-I, Jilin-j, Liaoning-L, Ningxia-N, Qinghai-Q, Shaanxi-a, Shandong-D, Shanxi-t and Tianjin-T). **b** Central Han (Anhui-A and Jiangsu-R), **c** South Han (Chongqing-C, Fujian-F, Guizhou-U, Hubei-W, Hunan-V, Jiangxi-n, Sichuan-d, Yunnan-Y and Zhejiang-Z), **d** Lingnan Han (Guangxi-E, Guangzhou-G and Hainan-H). **e** PC3 and PC4 analysis. **f** PCA of Chinese, JPT, and KHV individuals from the 1000 Genomes Projects. The blue "J" represents the JPT individuals, while "K" indicates the KHV individuals.

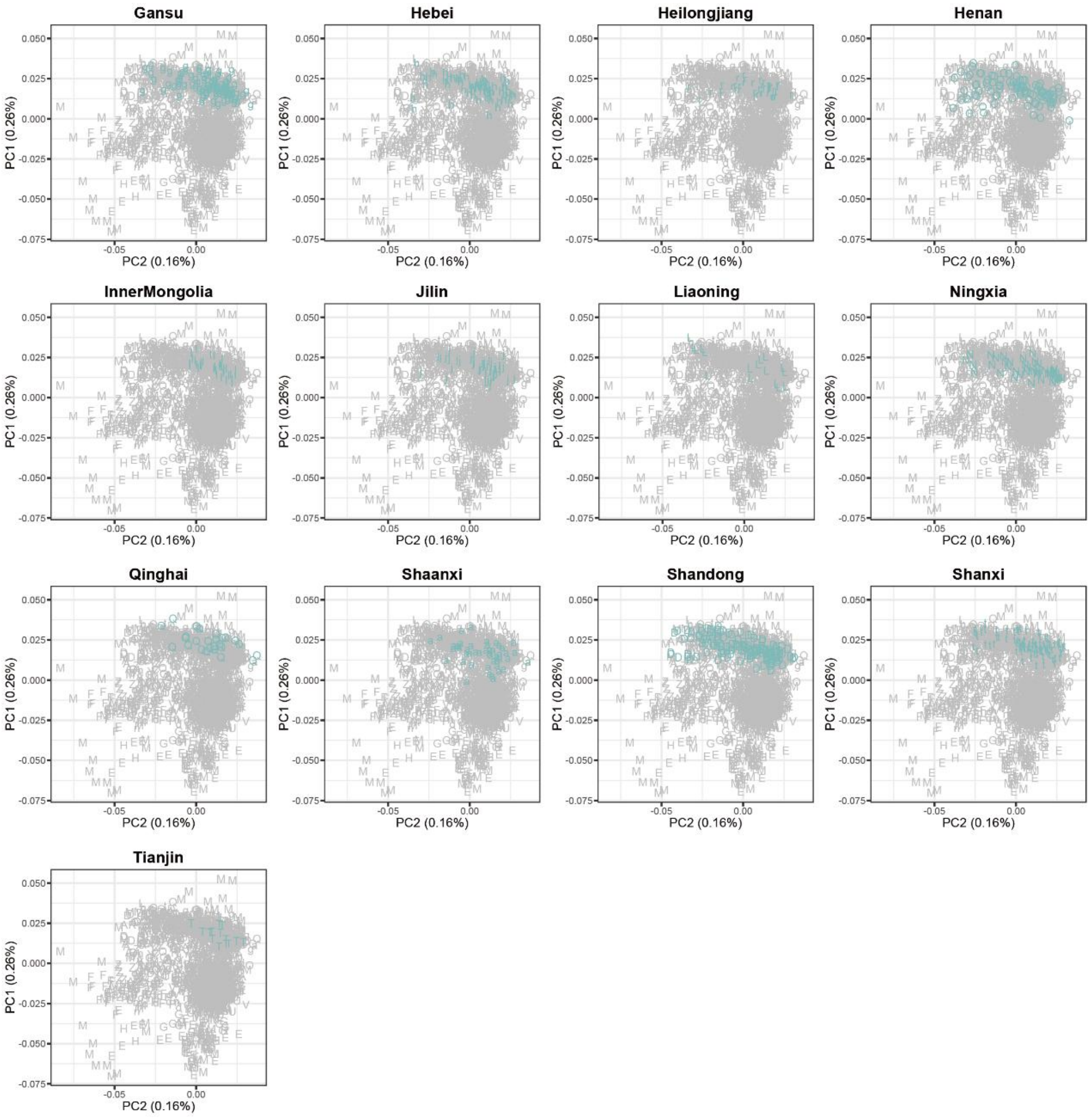

Supplementary Figure 5. PCA of North Han Chinese samples.

Each letter represents one administrative division. The individuals are highlighted in cyan color in north regions, including Gansu (g), Hebei (h), Heilongjiang (r), Henan (O), Inner Mongolia (I), Jilin (j), Liaoning (L), Ningxia (N), Qinghai (Q), Shaanxi (a), Shandong (D), Shanxi (t) and Tianjin (T).

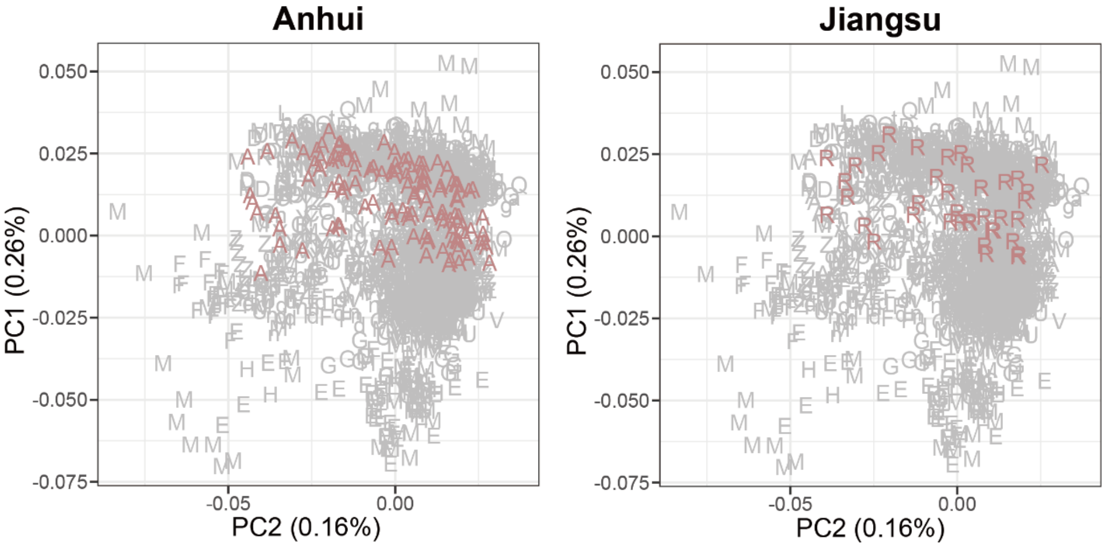

Supplementary Figure 6. PCA of Central Han Chinese samples.

Each letter represents one administrative division. The individuals are highlighted in dark-red for the central regions of Anhui (A) and Jiangsu (R).

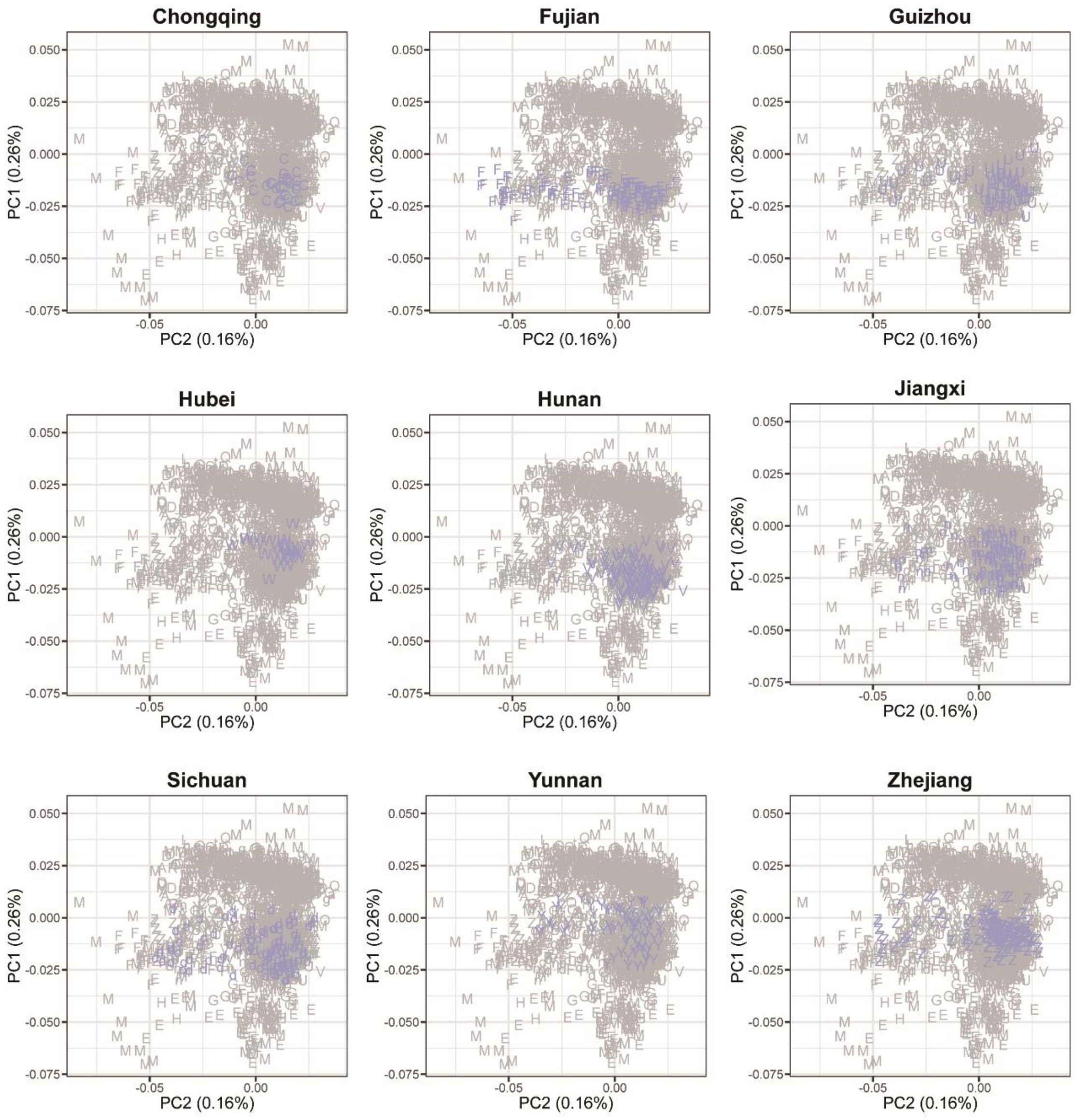

Supplementary Figure 7. PCA of South Han Chinese samples.

Each letter represents one administrative division. The individuals are highlighted in purple color for the southern regions of Chongqing (C), Fujian (F), Guizhou (U), Hubei (W), Hunan (V), Jiangxi (n), Sichuan (d), Yunnan (Y) and Zhejiang (Z).

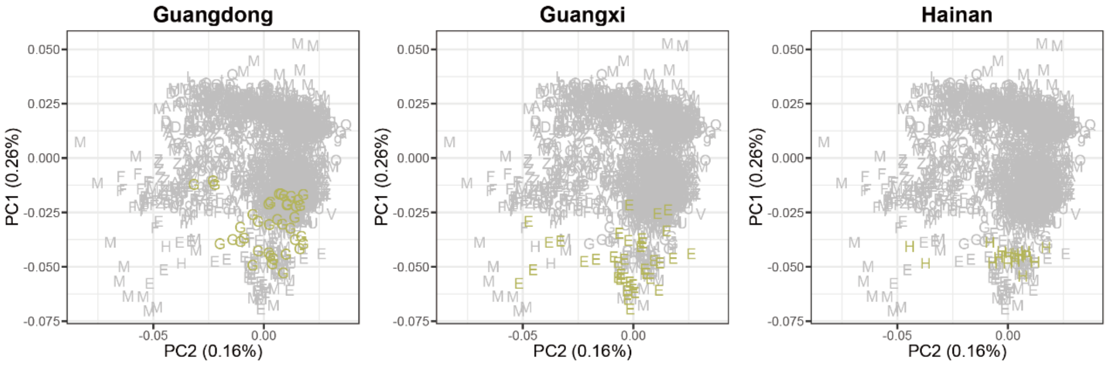

Supplementary Figure 8. PCA of Lingnan Han Chinese samples.

Each letter represents one administrative division. The individuals are highlighted in golden color for the Lingnan regions of Guangxi (E), Guangzhou (G) and Hainan (H).

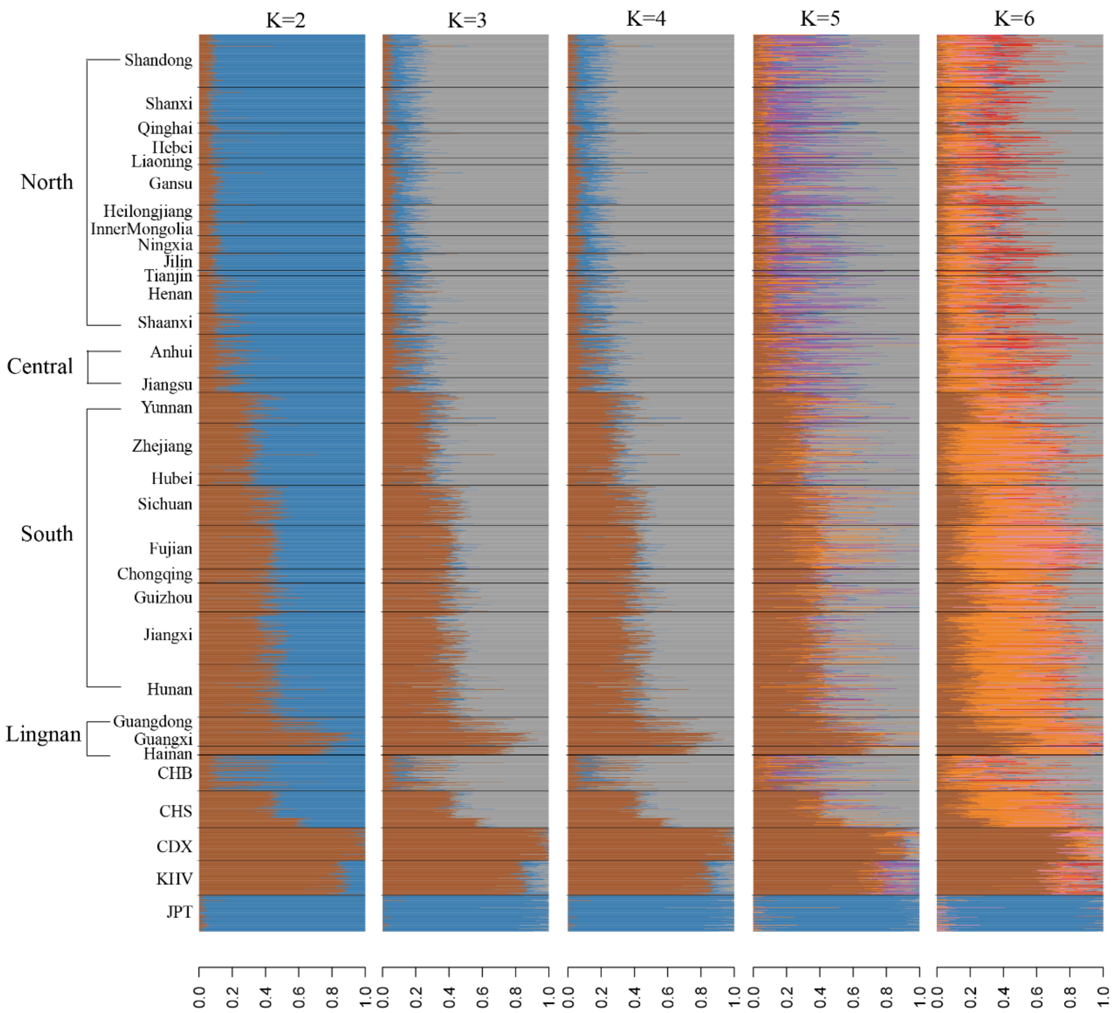

Supplementary Figure 9. ADMIXTURE analysis of the Han Chinese, CHB, CHS, CDX, KHV and JPT individuals from the 1000 Genomes Projects.

The Han Chinese (2,056 individuals) was selected from our WBBC cohort. CHB (103 individuals) is a Han Chinese in Beijing. CHS (105 individuals) is Han Chinese from southern China. CDX (93 individuals) is Chinese Dai in Xishuangbanna. KHV (99 individuals) is Vietnamese from Kinh in Ho Chi Minh City. JPT (104 individuals) is Japanese in Tokyo. The provinces and regions are shown on the left. At K = 4, the cross-validation error was the lowest. Source data are provided as a Source Data file.

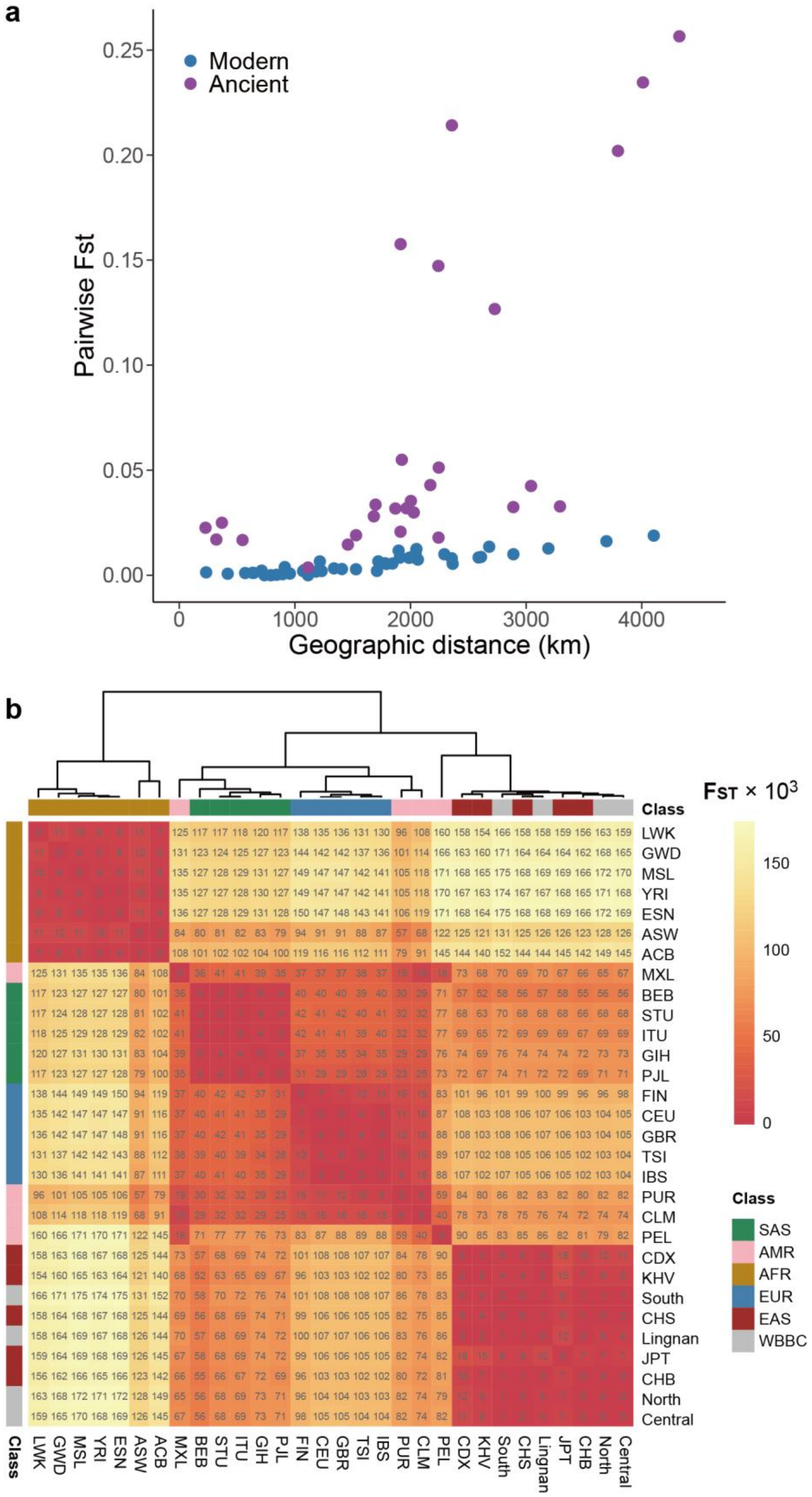
a
Modern
Ancient
Pairwise Fst
0.25
0.20
0.15
0.10
0.05
0.00
0
1000
2000
3000
4000
Geographic distance (km)
b
Class
FST × 10^3
150
100
50
0
LWK
GWD
MSL
YRI
ESN
ASW
ACB
MXL
BEB
STU
ITU
GIH
PJL
FIN
CEU
GBR
TSI
IBS
PUR
CLM
PEL
CDX
KHV
South
CHS
Lingnan
JPT
CHB
North
Central
Class
SAS
AMR
AFR
EUR
EAS
WBBC

Supplementary Figure 10. Pairwise Fst analysis.

a Relationship between pairwise Fst and geographic distances for the ancient and modern population. **b** Pairwise F_{ST} between the WBBC and 1KG Project populations. Numbers in each rectangle mean the pairwise F_{ST} value times 1,000. The bars on the top and left show the population classifications. SAS (South Asian), AMR (Admixed American), AFR (African), EUR (European), and EAS (East Asian) are five continent-level ancestry groups of the 1KG Project. CHB (Han Chinese in Beijing, China), JPT (Japanese in Tokyo, Japan), CHS (Southern Han Chinese), CDX (Chinese Dai in Xishuangbanna, China), KHV (Kinh in Ho Chi Minh City, Vietnam), CEU (Utah Residents (CEPH) with Northern and Western European Ancestry), TSI (Toscani in Italia),FIN(Finnish in Finland), GBR (British in England and Scotland), IBS (Iberian Population in Spain), YRI (Yoruba in Ibadan, Nigeria), LWK (Luhya in Webuye, Kenya), GWD (Gambian in Western Divisions in the Gambia), MSL (Mende in Sierra Leone), ESN (Esan in Nigeria), ASW (Americans of African Ancestry in SW USA), ACB (African Caribbeans in Barbados), MXL (Mexican Ancestry from Los Angeles USA), PUR (Puerto Ricans from Puerto Rico), CLM (Colombians from Medellin, Colombia), PEL (Peruvians from Lima, Peru), GIH (Gujarati Indian from Houston, Texas), PJL (Punjabi from Lahore, Pakistan), BEB (Bengali from Bangladesh), STU (Sri Lankan Tamil from the UK) and ITU (Indian Telugu from the UK). Source data are provided as a Source Data file.

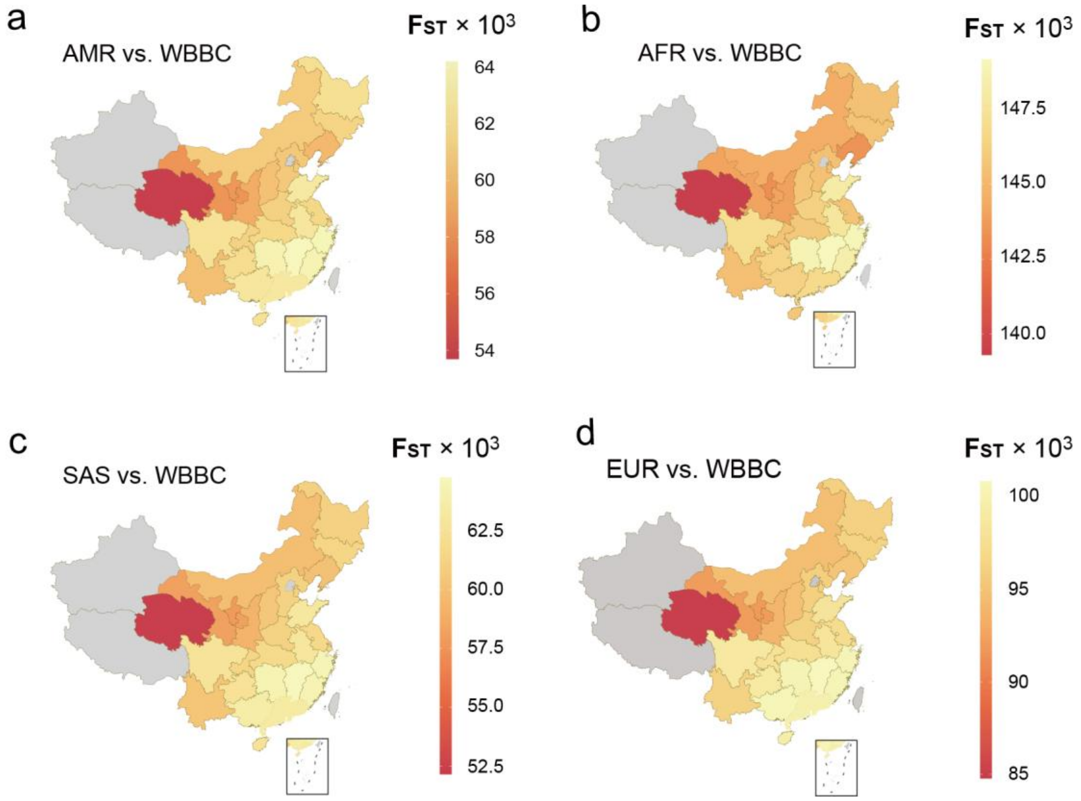

Supplementary Figure 11. Geographic patterns of pairwise F_{ST} using the 1KG as a reference.

Using the four non-Chinese continent-level ancestry groups of the 1KG Project as the reference, including **a** AMR. **b** AFR. **c** SAS. **d** EUR. We further investigated the geographic patterns of F_{ST} in the 27 administrative divisions. Regions in grey were not sampled.

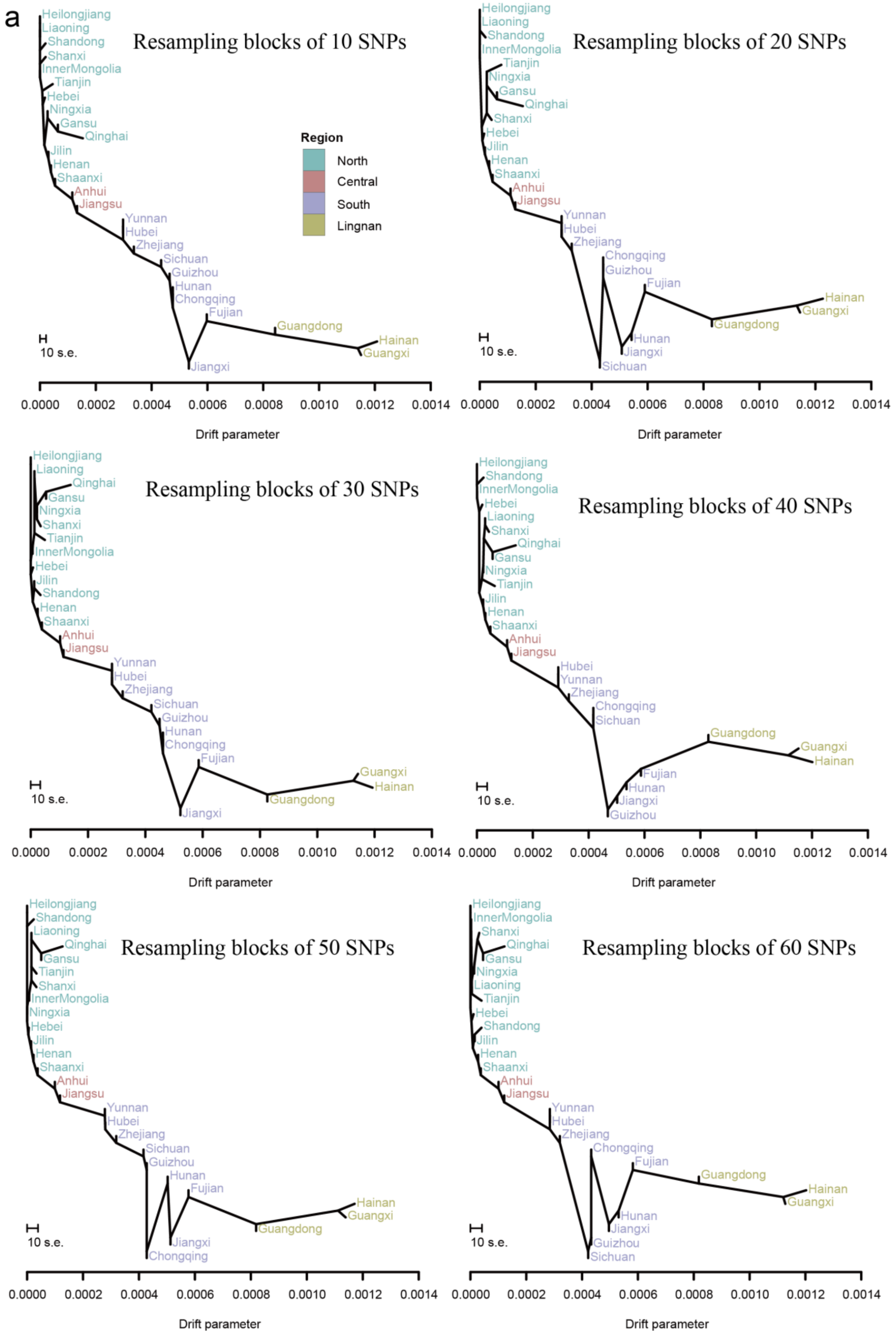

a
Resampling blocks of 10 SNPs
Heilongjiang
Liaoning
Shandong
Shanxi
InnerMongolia
Tianjin
Hebei
Ningxia
Gansu
Qinghai
Jilin
Henan
Shaanxi
Anhui
Jiangsu
Yunnan
Hubei
Zhejiang
Sichuan
Guizhou
Hunan
Chongqing
Fujian
Guangdong
Hainan
Guangxi
Jiangxi
10 s.e.
Region
North
Central
South
Lingnan
0.0000 0.0002 0.0004 0.0006 0.0008 0.0010 0.0012 0.0014
Drift parameter
Resampling blocks of 20 SNPs
Heilongjiang
Liaoning
Shandong
InnerMongolia
Tianjin
Ningxia
Gansu
Qinghai
Shanxi
Hebei
Jilin
Henan
Shaanxi
Anhui
Jiangsu
Yunnan
Hubei
Zhejiang
Chongqing
Guizhou
Fujian
Hainan
Guangxi
Guangdong
Hunan
Jiangxi
Sichuan
10 s.e.
0.0000 0.0002 0.0004 0.0006 0.0008 0.0010 0.0012 0.0014
Drift parameter
Resampling blocks of 30 SNPs
Heilongjiang
Liaoning
Qinghai
Gansu
Ningxia
Shanxi
Tianjin
InnerMongolia
Hebei
Jilin
Shandong
Henan
Shaanxi
Anhui
Jiangsu
Yunnan
Hubei
Zhejiang
Sichuan
Guizhou
Hunan
Chongqing
Fujian
Guangxi
Hainan
Guangdong
Jiangxi
10 s.e.
0.0000 0.0002 0.0004 0.0006 0.0008 0.0010 0.0012 0.0014
Drift parameter
Resampling blocks of 40 SNPs
Heilongjiang
Shandong
InnerMongolia
Hebei
Liaoning
Shanxi
Qinghai
Gansu
Ningxia
Tianjin
Jilin
Henan
Shaanxi
Anhui
Jiangsu
Hubei
Yunnan
Zhejiang
Chongqing
Sichuan
Guangdong
Guangxi
Hainan
Fujian
Hunan
Jiangxi
Guizhou
10 s.e.
0.0000 0.0002 0.0004 0.0006 0.0008 0.0010 0.0012 0.0014
Drift parameter
Resampling blocks of 50 SNPs
Heilongjiang
Shandong
Liaoning
Qinghai
Gansu
Tianjin
Shanxi
InnerMongolia
Ningxia
Hebei
Jilin
Henan
Shaanxi
Anhui
Jiangsu
Yunnan
Hubei
Zhejiang
Sichuan
Guizhou
Hunan
Fujian
Hainan
Guangxi
Guangdong
Jiangxi
Chongqing
10 s.e.
0.0000 0.0002 0.0004 0.0006 0.0008 0.0010 0.0012 0.0014
Drift parameter
Resampling blocks of 60 SNPs
Heilongjiang
InnerMongolia
Shanxi
Qinghai
Gansu
Ningxia
Liaoning
Tianjin
Hebei
Shandong
Jilin
Henan
Shaanxi
Anhui
Jiangsu
Yunnan
Hubei
Zhejiang
Chongqing
Fujian
Guangdong
Hainan
Guangxi
Hunan
Jiangxi
Guizhou
Sichuan
10 s.e.
0.0000 0.0002 0.0004 0.0006 0.0008 0.0010 0.0012 0.0014
Drift parameter

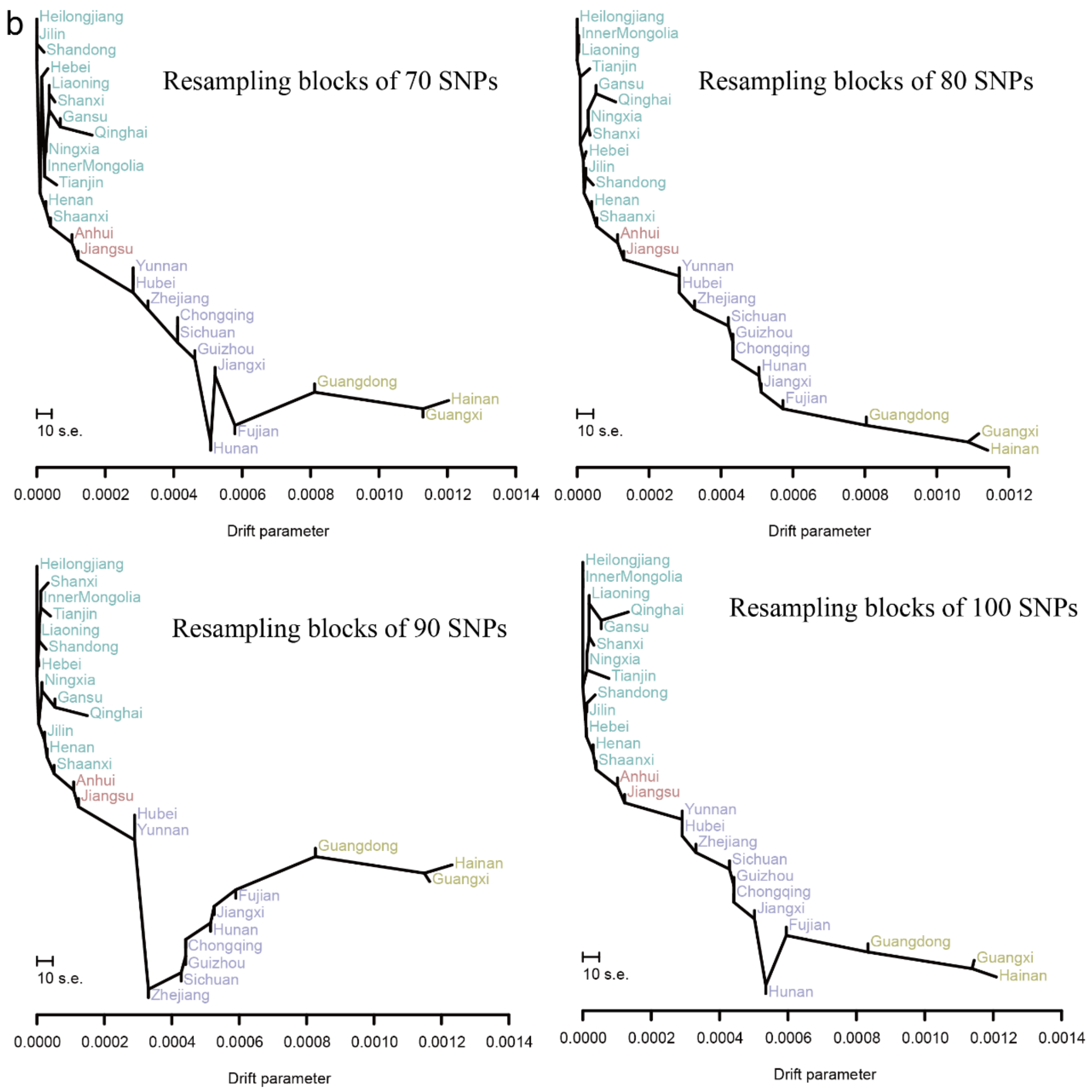

Supplementary Figure 12. Bootstrap replicates for the tree topology in 27 administrative divisions.

a and **b**, the bootstrap replicates were generated by the -bootstrap -k flag of TreeMix software. The plots were y-axis free. The scale bar shows ten times the average standard error of the entries in the sample covariance matrix for the estimated drift parameter.

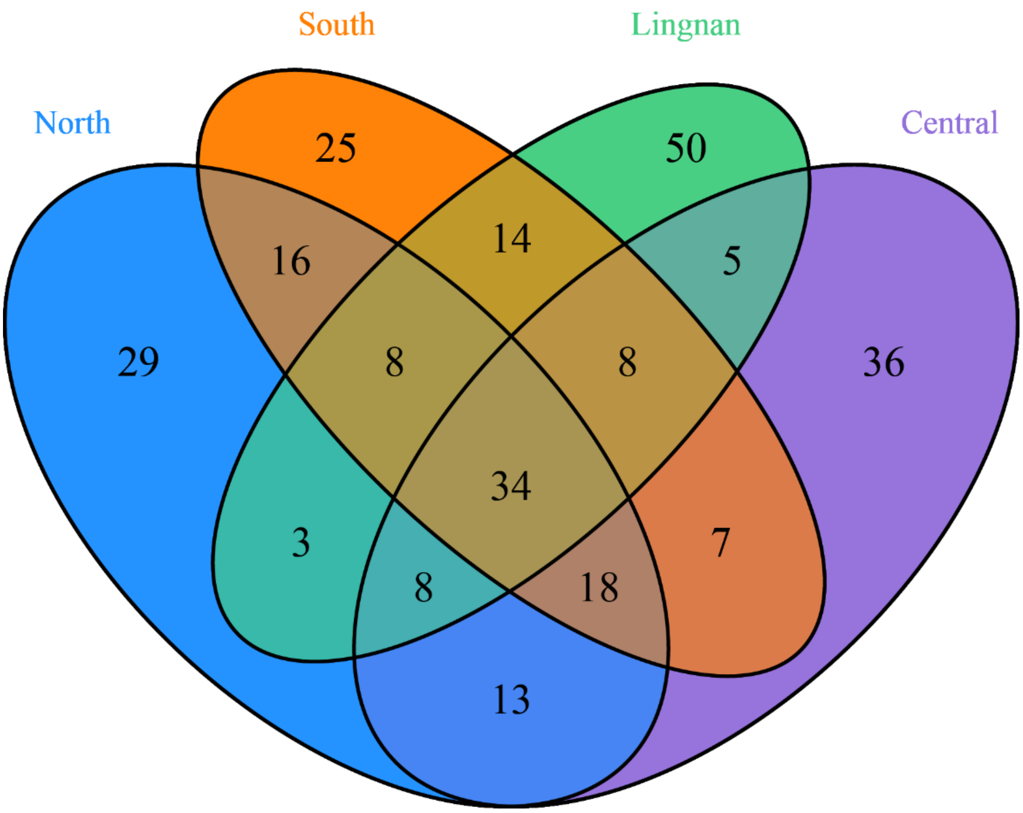

Supplementary Figure 13. The number of genomic regions with significant selection signatures.

Sharing of genomic regions with higher iHS signals among four subgroups. The number listed within the Venn Diagram indicates the numbers of 200 kb non-overlapping genomic windows in the top 1% of the fraction of SNVs with |iHS| >2.

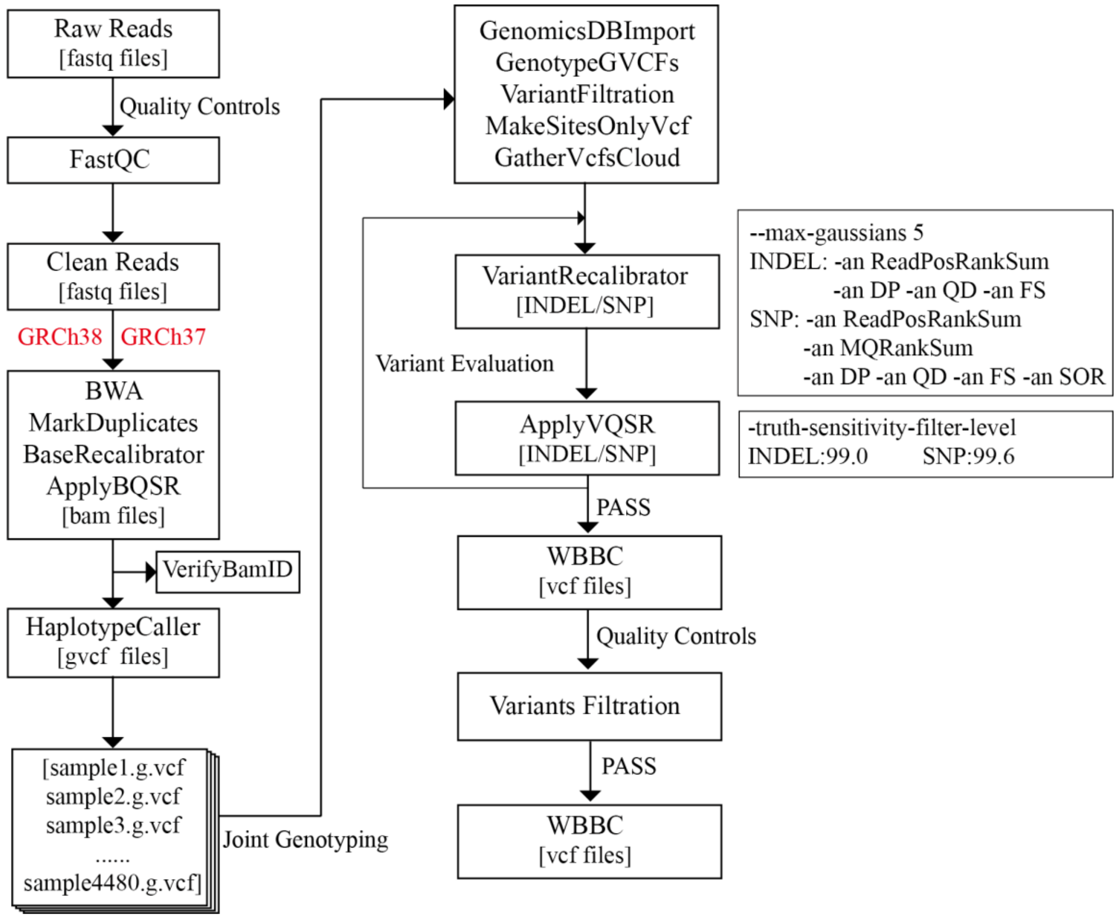

Supplementary Figure 14. Schematic representation of whole-genome sequencing analysis pipelines.

The BWA and GATK4 recommendation pipeline and variant analysis strategy were performed on the WBBC-cohort dataset with the GRCh38 and GRCh37 human reference genome, respectively.

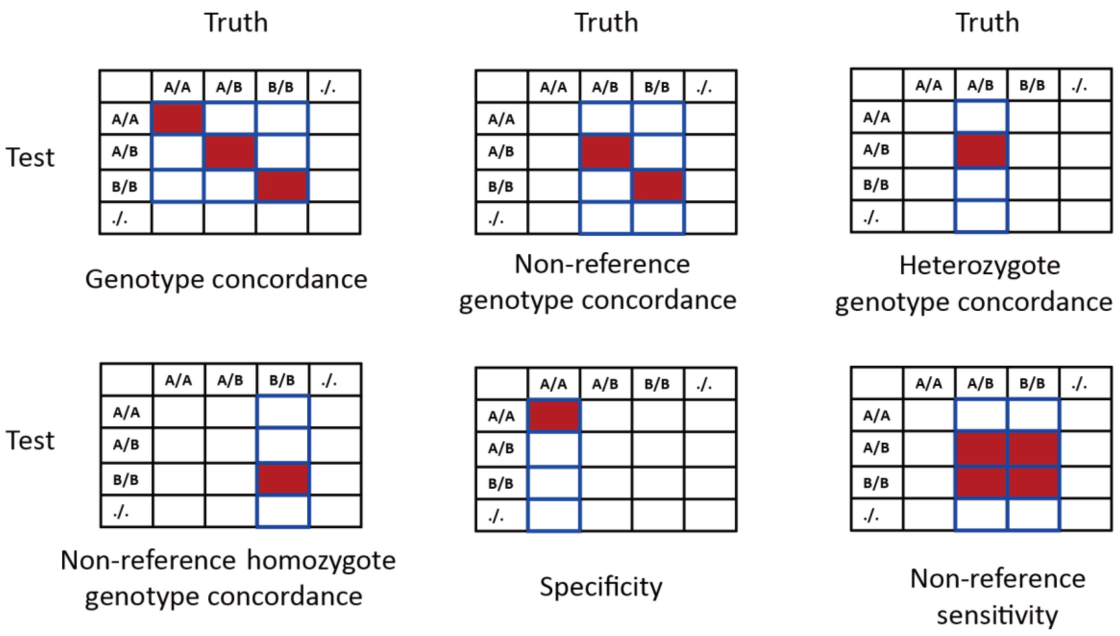

Supplementary Figure 15. Definition of genotype concordance, specificity, and sensitivity.

Letter A represents reference allele while B represents non-reference (NR) allele. The dot means missing called allele. For each metric, the value equals to the corresponding red rectangles divided by all rectangles with a blue border.

Supplementary Table 1. Geographic distribution of all the WBBC cohort samples. The individuals were recruited from 29 of the 34 administrative divisions of the People's Republic of China (PRC)

**Regions**	**Provinces**	**Number of samples for WGS**		**Number of samples for array**		**Total**
		**Male**	**Female**	**Male**	**Female**	
North (3,621)	Gansu	11	30	23	58	122
	Hebei	5	11	18	41	75
	Heilongjiang	7	6	15	25	53
	Henan	11	13	28	60	112
	Inner Mongolia	6	10	18	22	56
	Jilin	2	11	15	30	58
	Liaoning	1	0	6	17	24
	Ningxia	7	16	8	27	58
	Qinghai	5	17	10	13	45
	Shaanxi	7	15	12	25	59
	Shandong	8	12	1,066	1,735	2,821
	Shanxi	7	21	22	59	109
	Tianjin	0	0	9	7	16
	Tibet	0	1	0	0	1
	Xinjiang	4	5	1	2	12
Central (173)	Anhui	13	28	28	59	128
	Jiangsu	4	6	10	25	45
South (6,397)	Chongqing	3	12	8	20	43
	Fujian	6	51	17	58	132
	Guizhou	12	18	46	53	129
	Hubei	5	12	9	12	38
	Hunan	1,567	1,636	4	5	3,212
	Jiangxi	304	415	425	1,305	2,449
	Sichuan	13	26	26	57	122
	Yunnan	3	30	29	59	121
	Zhejiang	19	27	37	68	151
Lingnan (185)	Guangdong	8	18	9	14	49
	Guangxi	13	23	11	47	94
	Hainan	0	14	7	21	42
**Total**		2,051	2,484	1,917	3,924	**10,376**

Supplementary Table 2. Statistics of variants in 4,480 individuals

	MAF <0.5%	0.5%≤MAF≤5%	MAF >5%
**Variants type**			
SNVs	66,281,114	2,766,031	5,071,046
Novel SNVs (not in dbSNP151)	29,013,665	1,518	236
Novel SNVs (not in 1000G)	55,455,951	243,662	121,202
Indels	6,177,123	832,944	370,737
Novel INDELs (not in dbSNP151)	2,345,163	8,184	379
Novel INDELs (not in 1000G)	5,506,283	631,725	106,124
**Location**			
Exon	1,158,724	38,332	54,720
Intron	32,377,367	1,616,695	2,323,389
Splice-site	98,264	4,462	5,564
UTR	1,041,464	47,391	61,493
Upstream	508,527	23,373	33,849
Downsteam	499,095	25,464	35,601
Intergenic	36,774,796	1,843,258	2,927,167
**Function**			
Synonymous	307,745	11,172	17,632
Missense	529,851	13,450	15,372
Stoploss	658	21	16
Stopgain	13,825	184	131
frameshift insertion	3,687	96	48
frameshift deletion	8,803	142	70
nonframeshift insertion	2,503	132	53
nonframeshift deletion	6,855	210	104
**Disease-associated**			
SIFT:deleterious	249,592	5,142	3,221
Polyphen2:probably damaging	321,968	5,542	2,915
Polyphen2:possibly damaging	176,566	3,914	2,760
MutationTaster:disease causing	314,637	5,380	567
ClinVar:Pathogenic	1,273	19	14
ClinVar:Likely pathogenic	526	6	4

10bp from exon-intron boundary as splice variants

Supplementary Table 3. Average number of autosomal variants in each genome of four regions

	Han Chinese (n = 1151, Depth = 16.65 ×)		North (n = 213, Depth = 16.56 ×)		Central (n = 42, Depth = 16.67 ×)		South (n = 845, Depth = 16.66 ×)		Lingnan (n = 51, Depth = 16.89 ×)	
Annotation	No.	Het/Hom	No.	Het/Hom	No.	Het/Hom	No.	Het/Hom	No.	Het/Hom
**Type**										
SNVs	3,068,811	1.28	3,074,718	1.3	3,068,280	1.28	3,066,958	1.27	3,067,461	1.28
Novel SNVs (not in dbSNP151)	7,881	317.56	8,907	390.18	8,573	360.52	7,240	335.61	7,626	298.29
INDELs	257,832	1.51	258,507	1.52	258,496	1.5	258,973	1.5	257,560	1.51
Novel INDELs (not in dbSNP151)	1,607	8.64	1,656	9.1	1,648	8.95	1,605	8.55	1,593	8.52
**Location**										
Exon	32,411	1.35	32,473	1.37	32,398	1.35	32,355	1.34	32,400	1.35
Intron	1,417,225	1.3	1,419,904	1.32	1,417,090	1.3	1,416,690	1.3	1,416,589	1.3
Splice-site	3,269	1.35	3,273	1.37	3,265	1.35	3,266	1.35	3,269	1.34
UTR	36,785	1.32	36,872	1.34	36,856	1.32	36,787	1.32	36,760	1.31
Upstream	20,776	1.26	20,825	1.28	20,797	1.27	20,769	1.26	20,763	1.26
Downsteam	21,704	1.32	21,764	1.34	21,759	1.33	21,709	1.32	21,686	1.32
Intergenic	1,794,472	1.29	1,798,114	1.31	1,794,610	1.29	1,794,355	1.28	1,793,554	1.29
**Function**										
Synonymous	10,391	1.35	10,397	1.36	10,367	1.34	10,367	1.34	10,393	1.34
Missense	9,106	1.36	9,128	1.38	9,111	1.36	9,102	1.36	9,100	1.35
Stoploss	10	1.39	10	1.4	10	1.43	10	1.34	10	1.39
Stopgain	73	2.37	74	2.41	71	2.19	73	2.33	73	2.37
Frameshift insertion	28	1.63	28	1.64	28	1.66	27	1.61	27	1.62
Frameshift deletion	43	2.67	43	2.78	43	3.05	42	2.54	43	2.64

Non-frameshift insertion	52	1.04	53	1.06	53	1.06	54	1.1	52	1.03
Non-frameshift deletion	67	2.15	68	2.2	67	2.22	67	2.22	66	2.13
**Disease-associated**										
SIFT:deleterious	1,654	2.82	1,656	2.86	1,658	2.8	1,654	2.82	1,654	2.81
Polyphen2:probably damaging	968	3.28	971	3.35	971	3.22	964	3.2	968	3.28
Polyphen2:possibly damaging	771	2.86	773	2.89	771	2.82	769	2.83	771	2.85
MutationTaster:disease causing	8,769	1.24	8,794	1.26	8,772	1.25	8,753	1.24	8,763	1.24
ClinVar:Pathogenic	11	1.91	11	1.78	12	2.54	12	2.06	11	1.9

10bp from exon-intron boundary as splice variants

Supplementary Table 4. SNVs with significant selection signatures by SDS analysis in the Han Chinese population. Two-tailed *P*-values were calculated by whole genome-wide standardized SDS z-scores.

**Chr**	**chr3**	**chr4**	**chr4**	**chr4**	**chr4**	**chr4**	**chr4**	**chr6**	**chr12**	**chr12**	**chr12**	**chr12**	**chr16**
Position (GRCh38)	52351102	10140247	99318162	99079401	99279352	99212932	99134711	30595202	111803962	112379979	111730205	111672685	12563425
ID	rs78947691	rs148629931	rs1229984	rs1154413	rs3819197	rs2156733	rs4148887	rs9380181	rs671	rs11066280	rs11066015	rs3782886	rs75431978
Ancestral/derived allele	G/C	C/A	C/T	G/T	C/T	T/C	A/G	A/T	G/A	T/A	G/A	T/C	G/A
Derived allele frequency	0.270	0.058	0.710	0.726	0.790	0.764	0.734	0.151	0.239	0.265	0.240	0.242	0.181
Gene	*DNAH1*	*WDR1*	*ADH1B*	*ADH5*	*ADH1A*	*ADH6*	*ADH4*	MHC region	*ALDH2*	*HECTD4*	*ACAD10*	*BRAP*	*SNX29*
Function	Intron	upstream	Missense	Intron	Intron	Intron	Intron	Intergenic	Missense	Intron	Intron	Synonymous	Intron
NM	NM_015512.4	NM_017491.5	NM_000668.5	NM_000671.4	NM_000667.4	NM_000672.3	NM_000670.4	-	NM_000690.4	NM_001109662.3	NM_025247.5	NM_006768.4	NM_032167.4
cDNA	c.2729+512G>A	-	c.143A>G	c.344+1964C>A	c.1103+74G>A	c.262+674A>G	c.582+1755T>C	-	c.1510G>A	c.-256+1973A>T	c.1394+249G>A	c.723A>G	c.2319-5081G>A
Protein	-	-	p.His48Arg	-	-	-	-	-	p.Glu504Lys	-		p.Arg241Arg	-
Z-score	5.56	5.44	8.09	7.70	7.17	6.85	6.63	6.53	7.67	7.40	7.00	6.93	8.10
pValue	2.65E-08	5.44E-08	6.07E-16	1.38E-14	7.62E-13	7.59E-12	3.36E-11	6.43E-11	1.68E-14	1.41E-13	2.57E-12	4.11E-12	5.54E-16
DAF 1KG EAS	0.272	0.062	0.697	0.726	0.799	0.784	0.734	0.126	0.174	0.216	0.176	0.175	0.146
DAF 1KG EAS-CHB	0.214	0.039	0.709	0.689	0.791	0.757	0.689	0.078	0.160	0.189	0.160	0.155	0.175
DAF 1KG EAS-CHS	0.310	0.067	0.757	0.771	0.848	0.814	0.771	0.148	0.271	0.295	0.281	0.271	0.200
DAF 1KG EAS-CDX	0.355	0.081	0.634	0.737	0.753	0.758	0.731	0.258	0.043	0.134	0.043	0.043	0.113
DAF 1KG EAS-JPT	0.144	0.067	0.731	0.707	0.803	0.812	0.726	0.053	0.240	0.236	0.240	0.250	0.120
DAF 1KG EAS-KHV	0.348	0.056	0.646	0.727	0.793	0.773	0.753	0.106	0.136	0.217	0.136	0.136	0.116
DAF 1KG SAS	0.003	0.044	0.020	0.291	0.499	0.457	0.314	0.000	0.000	0.004	0.000	0.000	0.062
DAF 1KG EUR	0.120	0.000	0.029	0.141	0.255	0.260	0.130	0.000	0.000	0.000	0.000	0.000	0.003
DAF 1KG AMR	0.003	0.000	0.058	0.086	0.180	0.184	0.086	0.000	0.003	0.003	0.003	0.003	0.154
DAF 1KG AFR	0.000	0.001	0.002	0.011	0.250	0.479	0.014	0.000	0.002	0.001	0.000	0.000	0.002
DAF 1KG All	0.058	0.021	0.159	0.246	0.400	0.451	0.251	0.025	0.036	0.045	0.036	0.036	0.064

Supplementary Table 5. Pair-wise Fst values for 27 provinces of China in WBBC

**Provinces**	**He bei**	**Shan xi**	**Inner Mongolia**	**Liao ning**	**Ji lin**	**Hei Long jiang**	**Shan dong**	**He nan**	**Jiang su**	**Zhe jiang**	**An hui**	**Fu jian**	**Jiang xi**	**Hu bei**	**Hu nan**	**Guang dong**	**Guang xi**	**Hai nan**	**Chong qing**	**Si chuan**	**Gui zhou**	**Yun nan**	**Shaan xi**	**Gan su**	**Qing hai**	**Ning xia**	**Tian jin**
**Hebei**	0.00	0.04	0.06	0.00	0.07	0.00	0.14	0.02	0.14	1.12	0.20	2.42	2.01	0.82	1.96	4.39	6.22	6.49	1.53	1.66	1.89	1.21	0.20	0.23	0.92	0.12	0.24
**Shanxi**	0.04	0.00	0.00	0.00	0.05	0.00	0.11	0.05	0.32	1.33	0.29	2.63	2.23	0.99	2.25	4.58	6.76	7.02	1.72	1.91	2.14	1.28	0.12	0.08	0.65	0.00	0.11
**InnerMongolia**	0.06	0.00	0.00	0.00	0.00	0.00	0.11	0.02	0.27	1.14	0.27	2.42	1.94	0.69	1.89	4.26	6.35	6.25	1.21	1.66	1.98	1.14	0.15	0.12	0.68	0.10	0.09
**Liaoning**	0.00	0.00	0.00	0.00	0.00	0.00	0.00	0.00	0.27	1.06	0.07	2.21	1.93	0.88	1.83	4.35	6.47	6.56	1.10	1.49	1.79	1.02	0.00	0.00	0.36	0.00	0.05
**Jilin**	0.07	0.05	0.00	0.00	0.00	0.00	0.00	0.00	0.01	1.03	0.10	2.14	1.74	0.67	1.79	4.03	6.27	6.51	1.38	1.45	1.69	1.10	0.16	0.20	0.87	0.18	0.36
**Heilongjiang**	0.00	0.00	0.00	0.00	0.00	0.00	0.00	0.00	0.13	1.01	0.07	2.20	1.87	0.68	1.96	4.19	6.32	6.54	1.53	1.51	1.90	1.11	0.06	0.04	0.77	0.06	0.23
**Shandong**	0.14	0.11	0.11	0.00	0.00	0.00	0.00	0.02	0.16	1.19	0.22	2.39	2.05	0.99	2.17	4.39	6.56	6.87	1.70	1.83	2.10	1.35	0.29	0.42	1.22	0.30	0.36
**Henan**	0.02	0.05	0.02	0.00	0.00	0.00	0.02	0.00	0.15	0.92	0.13	1.96	1.63	0.64	1.68	3.83	5.71	5.99	1.36	1.34	1.69	1.07	0.18	0.22	0.88	0.13	0.30
**Jiangsu**	0.14	0.32	0.27	0.27	0.01	0.13	0.16	0.15	0.00	0.44	0.00	1.32	1.10	0.24	1.10	2.99	4.69	4.96	0.87	0.95	1.23	0.69	0.31	0.50	1.09	0.33	0.25
**Zhejiang**	1.12	1.33	1.14	1.06	1.03	1.01	1.19	0.92	0.44	0.00	0.46	0.51	0.24	0.11	0.32	1.63	2.90	3.14	0.38	0.36	0.56	0.54	1.00	1.40	2.14	1.20	1.08
**Anhui**	0.20	0.29	0.27	0.07	0.10	0.07	0.22	0.13	0.00	0.46	0.00	1.51	1.08	0.37	1.17	3.13	4.87	5.17	0.91	0.99	1.21	0.76	0.22	0.42	1.21	0.32	0.31
**Fujian**	2.42	2.63	2.42	2.21	2.14	2.20	2.39	1.96	1.32	0.51	1.51	0.00	0.44	0.81	0.51	1.30	2.02	2.03	0.83	0.43	0.71	0.96	2.16	2.57	3.22	2.26	2.77
**Jiangxi**	2.01	2.23	1.94	1.93	1.74	1.87	2.05	1.63	1.10	0.24	1.08	0.44	0.00	0.34	0.05	0.98	1.87	2.13	0.30	0.28	0.42	0.72	1.76	2.22	2.92	1.93	1.90
**Hubei**	0.82	0.99	0.69	0.88	0.67	0.68	0.99	0.64	0.24	0.11	0.37	0.81	0.34	0.00	0.22	1.79	3.09	3.26	0.28	0.33	0.45	0.45	0.61	0.95	1.41	0.85	0.84
**Hunan**	1.96	2.25	1.89	1.83	1.79	1.96	2.17	1.68	1.10	0.32	1.17	0.51	0.05	0.22	0.00	0.81	1.56	1.82	0.11	0.09	0.17	0.53	1.67	2.13	2.83	1.85	2.14
**Guangdong**	4.39	4.58	4.26	4.35	4.03	4.19	4.39	3.83	2.99	1.63	3.13	1.30	0.98	1.79	0.81	0.00	0.64	0.75	1.30	1.31	1.22	1.88	3.70	4.29	5.07	4.01	4.38
**Guangxi**	6.22	6.76	6.35	6.47	6.27	6.32	6.56	5.71	4.69	2.90	4.87	2.02	1.87	3.09	1.56	0.64	0.00	0.37	2.10	1.77	1.73	2.66	5.61	6.37	6.87	6.08	6.80
**Hainan**	6.49	7.02	6.25	6.56	6.51	6.54	6.87	5.99	4.96	3.14	5.17	2.03	2.13	3.26	1.82	0.75	0.37	0.00	2.44	2.10	2.24	3.07	6.00	6.46	7.21	6.21	7.19
**Chongqing**	1.53	1.72	1.21	1.10	1.38	1.53	1.70	1.36	0.87	0.38	0.91	0.83	0.30	0.28	0.11	1.30	2.10	2.44	0.00	0.07	0.10	0.21	1.12	1.54	2.02	1.48	1.72
**Sichuan**	1.66	1.91	1.66	1.49	1.45	1.51	1.83	1.34	0.95	0.36	0.99	0.43	0.28	0.33	0.09	1.31	1.77	2.10	0.07	0.00	0.06	0.30	1.40	1.69	2.29	1.58	1.94
**Guizhou**	1.89	2.14	1.98	1.79	1.69	1.90	2.10	1.69	1.23	0.56	1.21	0.71	0.42	0.45	0.17	1.22	1.73	2.24	0.10	0.06	0.00	0.38	1.45	1.92	2.36	1.78	2.15
**Yunnan**	1.21	1.28	1.14	1.02	1.10	1.11	1.35	1.07	0.69	0.54	0.76	0.96	0.72	0.45	0.53	1.88	2.66	3.07	0.21	0.30	0.38	0.00	0.90	1.05	1.38	0.98	1.43
**Shaanxi**	0.20	0.12	0.15	0.00	0.16	0.06	0.29	0.18	0.31	1.00	0.22	2.16	1.76	0.61	1.67	3.70	5.61	6.00	1.12	1.40	1.45	0.90	0.00	0.06	0.61	0.17	0.27
**Gansu**	0.23	0.08	0.12	0.00	0.20	0.04	0.42	0.22	0.50	1.40	0.42	2.57	2.22	0.95	2.13	4.29	6.37	6.46	1.54	1.69	1.92	1.05	0.06	0.00	0.23	0.00	0.19
**Qinghai**	0.92	0.65	0.68	0.36	0.87	0.77	1.22	0.88	1.09	2.14	1.21	3.22	2.92	1.41	2.83	5.07	6.87	7.21	2.02	2.29	2.36	1.38	0.61	0.23	0.00	0.43	0.36
**Ningxia**	0.12	0.00	0.10	0.00	0.18	0.06	0.30	0.13	0.33	1.20	0.32	2.26	1.93	0.85	1.85	4.01	6.08	6.21	1.48	1.58	1.78	0.98	0.17	0.00	0.43	0.00	0.23
**Tianjin**	0.24	0.11	0.09	0.05	0.36	0.23	0.36	0.30	0.25	1.08	0.31	2.77	1.90	0.84	2.14	4.38	6.80	7.19	1.72	1.94	2.15	1.43	0.27	0.19	0.36	0.23	0.00

Supplementary Table 6. Pair-wise Fst values for 26 populations in 1KG Phase3 and four regions of China in WBBC

Regions	CHB	JPT	CHS	CDX	KHV	CEU	TSI	FIN	GBR	IBS	YRI	LWK	GWD	MSL	ESN	ASW	ACB	MXL	PUR	CLM	PEL	GIH	PJL	BEB	STU	ITU	North	South	Central	Lingnan
CHB	0	7	1.3	9.5	6.8	103.2	102.4	96.1	103.3	102.4	165.2	156	161.8	166.4	166.1	122.8	142	65.7	79.6	72.4	81.3	71.5	69.1	54.8	65.9	67.2	0.3	0.5	0.1	3.2
JPT	7	0	9.4	18.4	14.9	106.3	105.4	98.8	106.3	105.3	167.8	158.6	164.4	169.1	168.8	125.6	144.7	67.2	82	74.4	82.3	73.8	71.6	57.6	68.1	69.4	6.9	8.3	7.2	11.7
CHS	1.3	9.4	0	5.4	3.7	106.2	105.3	99.1	106.3	105.2	167.3	158.2	163.9	168.5	168.2	125.2	144.2	68.9	82.4	75.4	84.5	73.6	71.3	56.5	67.7	69.1	2.5	0.2	1.7	0.8
CDX	9.5	18.4	5.4	0	2.3	107.7	106.8	101.1	107.9	106.7	166.8	157.5	163.4	167.9	167.5	125.1	143.7	72.6	84.1	77.7	89.6	74.2	71.9	56.8	68	69.5	12	6.4	10.6	2.7
KHV	6.8	14.9	3.7	2.3	0	102.9	101.9	96.4	102.9	101.9	163.5	154.3	160.2	164.6	164.3	121.5	140.3	68	79.7	73.3	85.4	69.3	67.2	52.3	63.3	64.7	9	4.4	7.7	1.9
CEU	103.2	106.3	106.2	107.7	102.9	0	3.9	6.7	0.3	2.6	146.6	135.4	142	147.1	147.3	91.3	115.8	37	11.4	16.3	87.5	35.1	29.2	40.2	41.4	40.7	103.6	108	105	106.9
TSI	102.4	105.4	105.3	106.8	101.9	3.9	0	12.7	4.1	1.6	142.1	130.9	137.5	142.5	142.8	88.2	112	37.7	10.1	16	89.3	33.7	28	39	39.6	38.8	103.2	107.5	104.2	106
FIN	96.1	98.8	99.1	101.1	96.4	6.7	12.7	0	7.3	11	148.9	137.9	144.5	149.4	149.6	94.1	118.5	36.7	16.5	19.4	83.5	36.6	31.1	40	42.1	41.6	96.1	100.5	97.7	100
GBR	103.3	106.3	106.3	107.9	102.9	0.3	4.1	7.3	0	2.6	146.8	135.6	142.3	147.3	147.6	91.5	116	37.1	11.6	16.4	87.7	35.3	29.3	40.4	41.6	40.9	103.6	108	105.1	107
IBS	102.4	105.3	105.2	106.7	101.9	2.6	1.6	11	2.6	0	140.6	129.6	136	141	141.4	86.7	110.5	36.8	9	14.8	88.5	35.5	29.5	40.1	41.2	40.4	103.2	107.5	104.1	105.9
YRI	165.2	167.8	167.3	166.8	163.5	146.6	142.1	148.9	146.8	140.6	0	7.7	6.5	4.1	1	10.1	3	135.3	105.2	118	169.9	130.5	127.2	127	127.4	128.3	171.5	174.1	168.3	167.4
LWK	156	158.6	158.2	157.5	154.3	135.4	130.9	137.9	135.6	129.6	7.7	0	11.1	9.8	8.1	10.7	6.8	124.9	95.7	107.9	160.2	120.1	116.6	116.7	117.2	118	162.8	165.6	159.3	158.3
GWD	161.8	164.4	163.9	163.4	160.2	142	137.5	144.5	142.3	136	6.5	11.1	0	3.9	7.9	11.6	6.5	131.1	101.3	113.9	166	126.6	123.3	123.2	123.6	124.5	168.3	171	165	164
MSL	166.4	169.1	168.5	167.9	164.6	147.1	142.5	149.4	147.3	141	4.1	9.8	3.9	0	5.5	11.4	4.9	135.5	105.3	118.2	170.8	130.8	127.4	127.2	127.6	128.5	172.2	174.8	169.5	168.7
ESN	166.1	168.8	168.2	167.5	164.3	147.3	142.8	149.6	147.6	141.4	1	8.1	7.9	5.5	0	10.8	3.8	136	105.8	118.6	170.8	131	127.7	127.5	127.9	128.8	172	174.7	169.1	168.3
ASW	122.8	125.6	125.2	125.1	121.5	91.3	88.2	94.1	91.5	86.7	10.1	10.7	11.6	11.4	10.8	0	2.9	83.9	57.3	67.6	121.9	82.5	78.7	79.8	81.1	81.7	127.9	130.9	125.9	125.6
ACB	142	144.7	144.2	143.7	140.3	115.8	112	118.5	116	110.5	3	6.8	6.5	4.9	3.8	2.9	0	108.1	78.8	90.8	144.8	103.9	100.2	100.9	101.7	102.4	148.7	151.6	145.3	144.4
MXL	65.7	67.2	68.9	72.6	68	37	37.7	36.7	37.1	36.8	135.3	124.9	131.1	135.5	136	83.9	108.1	0	19.4	9.6	18.4	39.2	35	36.5	41.1	40.9	65.5	69.6	67	70.2
PUR	79.6	82	82.4	84.1	79.7	11.4	10.1	16.5	11.6	9	105.2	95.7	101.3	105.3	105.8	57.3	78.8	19.4	0	5.8	58.9	28.5	23.4	29.8	31.9	31.6	81.7	85.7	81.7	83.2
CLM	72.4	74.4	75.4	77.7	73.3	16.3	16	19.4	16.4	14.8	118	107.9	113.9	118.2	118.6	67.6	90.8	9.6	5.8	0	40.5	29.3	24.7	29.4	32.3	32.1	73.6	77.7	74.2	76.2
PEL	81.3	82.3	84.5	89.6	85.4	87.5	89.3	83.5	87.7	88.5	169.9	160.2	166	170.8	170.8	121.9	144.8	18.4	58.9	40.5	0	76.2	73.4	70.6	76.6	76.6	79.4	83.4	81.8	86
GIH	71.5	73.8	73.6	74.2	69.3	35.1	33.7	36.6	35.3	35.5	130.5	120.1	126.6	130.8	131	82.5	103.9	39.2	28.5	29.3	76.2	0	3.6	4.6	4.4	3.8	73	76.2	73.1	73.9
PJL	69.1	71.6	71.3	71.9	67.2	29.2	28	31.1	29.3	29.5	127.2	116.6	123.3	127.4	127.7	78.7	100.2	35	23.4	24.7	73.4	3.6	0	4	3.8	3.5	70.6	73.9	70.9	71.7
BEB	54.8	57.6	56.5	56.8	52.3	40.2	39	40	40.4	40.1	127	116.7	123.2	127.2	127.5	79.8	100.9	36.5	29.8	29.4	70.6	4.6	4	0	2.3	2.4	56	58.3	56.3	56.6

STU	65.9	68.1	67.7	68	63.3	41.4	39.6	42.1	41.6	41.2	127.4	117.2	123.6	127.6	127.9	81.1	101.7	41.1	31.9	32.3	76.6	4.4	3.8	2.3	0	1.3	67.7	70.3	67.6	67.9
ITU	67.2	69.4	69.1	69.5	64.7	40.7	38.8	41.6	40.9	40.4	128.3	118	124.5	128.5	128.8	81.7	102.4	40.9	31.6	32.1	76.6	3.8	3.5	2.4	1.3	0	68.9	71.7	68.9	69.4
North	0.3	6.9	2.5	12	9	103.6	103.2	96.1	103.6	103.2	171.5	162.8	168.3	172.2	172	127.9	148.7	65.5	81.7	73.6	79.4	73	70.6	56	67.7	68.9	0	1.5	0.2	5.1
South	0.5	8.3	0.2	6.4	4.4	108	107.5	100.5	108	107.5	174.1	165.6	171	174.8	174.7	130.9	151.6	69.6	85.7	77.7	83.4	76.2	73.9	58.3	70.3	71.7	1.5	0	0.8	1.3
Central	0.1	7.2	1.7	10.6	7.7	105	104.2	97.7	105.1	104.1	168.3	159.3	165	169.5	169.1	125.9	145.3	67	81.7	74.2	81.8	73.1	70.9	56.3	67.6	68.9	0.2	0.8	0	3.9
Lingnan	3.2	11.7	0.8	2.7	1.9	106.9	106	100	107	105.9	167.4	158.3	164	168.7	168.3	125.6	144.4	70.2	83.2	76.2	86	73.9	71.7	56.6	67.9	69.4	5.1	1.3	3.9	0

Supplementary Table 7. Pairwise Values of Genetic Distance (Fst) within modern and ancient populations

Populations	JPT	IMG	CHB	CHS	CDX	North	South	Central	Lingnan	KHV	ancient_ Japan	ancient_ China_ IMG	ancient_ China_ north	ancient_ China_ south	ancient_ China_ Taiwan	Ancient _Laos	ancient_ Thailand	ancient_ vietnam
**JPT**	-		-	-	-	-	-	-	-	-	-	-	-	-	-	-	-	-
**IMG**	0.0085	-	-	-	-	-	-	-	-	-	-	-	-	-	-	-	-	-
**CHB**	0.0081	0.0011	-	-	-	-	-	-	-	-	-	-	-	-	-	-	-	-
**CHS**	0.0100	0.0028	0.0025	-	-	-	-	-	-	-	-	-	-	-	-	-	-	-
**CDX**	0.0188	0.0135	0.0125	0.0056	-	-	-	-	-	-	-	-	-	-	-	-	-	-
**North**	0.0084	0.0000	0.0014	0.0017	0.0117	-	-	-	-	-	-	-	-	-	-	-	-	-
**South**	0.0100	0.0020	0.0021	0.0011	0.0066	0.0020	-	-	-	-	-	-	-	-	-	-	-	-
**Central**	0.0074	0.0000	0.0000	0.0007	0.0103	0.0005	0.0002	-	-	-	-	-	-	-	-	-	-	-
**Lingnan**	0.0127	0.0054	0.0054	0.0008	0.0020	0.0057	0.0018	0.0032	-	-	-	-	-	-	-	-	-	-
**KHV**	0.0162	0.0087	0.0083	0.0029	0.0022	0.0084	0.0039	0.0066	0.0010	-	-	-	-	-	-	-	-	-
**ancient_Japan**	-	-	-	-	-	-	-	-	-	-	-	-	-	-	-	-	-	-
**ancient_China_IMG**	-	-	-	-	-	-	-	-	-	-	0.1576	-	-	-	-	-	-	-
**ancient_China_north**	-	-	-	-	-	-	-	-	-	-	0.1267	0.0036	-	-	-	-	-	-
**ancient_China_south**	-	-	-	-	-	-	-	-	-	-	0.2142	0.0318	0.0190	-	-	-	-	-
**ancient_China_Taiwan**	-	-	-	-	-	-	-	-	-	-	0.1472	0.0429	0.0318	0.0250	-	-	-	-
**ancient_Laos**	-	-	-	-	-	-	-	-	-	-	0.2346	0.0425	0.0299	0.0280	0.0550	-	-	-
**ancient_Thailand**	-	-	-	-	-	-	-	-	-	-	0.2566	0.0327	0.0179	0.0353	0.0512	0.0170	-	-
**ancient_vietnam**	-	-	-	-	-	-	-	-	-	-	0.2020	0.0324	0.0207	0.0146	0.0336	0.0167	0.0226	-

Supplementary Table 8. Pair-wise Fst values for 27 provinces of China in WBBC and four continent groups in 1KG Phase3

Provinces	EAS_fst	EUR_fst	AFR_fst	AMR_fst	SAS_fst	location
**Beijing**	2.962	95.526	145.45	61.971	60.866	North
**Tianjing**	2.962	95.526	145.45	61.971	60.866	North
**Hebei**	2.962	95.526	145.45	61.971	60.866	North
**Shanxi**	3.2363	94.566	145.66	61.304	60.38	North
**Inner Mongolia**	2.8335	94.684	144.52	60.937	59.99	North
**Liaoning**	2.822	95.075	143.41	60.437	59.932	North
**Jilin**	2.7535	96.393	145.61	62.226	61.512	North
**Heilongjiang**	2.8901	96.251	145.51	62.33	61.331	North
**Shandong**	3.1539	98.123	148.22	64.34	63.235	North
**Henan**	2.6611	96.141	146.29	62.648	61.302	North
**Shaaxi**	2.6261	93.75	143.99	60.341	59.289	North
**Gansu**	3.0016	92.022	144.06	59.46	58.245	North
**Qinghai**	3.5344	86.774	139.31	54.621	53.693	North
**Ningxia**	2.8297	92.572	143.25	59.462	58.332	North
**Shanghai**	2.1319	97.181	146.08	63.186	61.562	Central
**Anhui**	2.1593	98.065	147.75	64.382	62.854	Central
**Jiangsu**	2.1119	97.159	145.59	63.149	61.698	Central
**Zhejiang**	1.2548	99.86	148.96	66.191	64.228	South
**Fujiang**	1.0677	100.01	148.41	66.524	64.053	South
**Jiangxi**	0.89921	100.42	149.19	66.771	64.477	South
**Hubei**	1.2102	97.319	145.79	63.111	61.741	South
**Hunan**	0.71417	99.663	148.83	66.256	63.913	South
**Chongqing**	0.8206	97.673	145.63	63.728	61.397	South
**Sichuan**	0.73752	98.337	147.07	65.061	62.526	South
**Guizhou**	0.74674	98.053	146.46	64.546	61.99	South
**Yunnan**	1.0233	96.188	145.4	62.915	60.378	South
**Guangdong**	0.65153	99.46	146.77	65.797	62.978	Lingnan
**Guangxi**	1.1767	99.534	146.34	66.443	62.834	Lingnan
**Hainan**	1.5023	99.407	145.74	65.637	62.474	Lingnan

Supplementary Table 9. GO (Gene Ontology) analysis of candidate genes in top 1% genomic regions with positive selection by iHS statistic

GO term	Categories	North	Central	South	Lingnan
GO biological process					
GO:0006067	ethanol metabolic process	0.001023	0.0154944	0.0022526	0.0005998
GO:0006069	ethanol oxidation	0.0008038	0.0154944	0.0018229	0.0000114
GO:0008544	epidermis development	-	-	0.0000594	0.0008144
GO:0009913	epidermal cell differentiation	-	-	0.0000091	0.0003389
GO:0016999	antibiotic metabolic process	-	0.0154944	0.0008874	-
GO:0017000	antibiotic biosynthetic process	-	0.0045203	0.0001621	-
GO:0030216	keratinocyte differentiation	-	-	0.0000079	0.0000755
GO:0031424	keratinization	-	-	0.0000002	0.0000073
GO:0043588	skin development	-	-	0.000079	0.000152
GO:0050665	hydrogen peroxide biosynthetic process	-	0.0096893	0.0007227	-
GO cellular component					
GO:0005882	intermediate filament	-	-	0.0000001	0.0000004
GO:0045095	keratin filament	-	-	0.0000079	0.0000755
GO:0045111	intermediate filament cytoskeleton	-	-	0.0000002	0.0000003

Benjamini and Hochberg (BH) correction was applied to enrichment *p* values for multiple testing. “-” represents that the adjusted *p*-value is >0.05.

Supplementary Table 10. KEGG (Kyoto Encyclopedia of Genes and Genomes) enrichment pathway for candidate genes in top 1% genomic regions with positive selection by iHS statistic

ID	Description	North	Central	South	Lingnan
hsa05034	Alcoholism	0.003459	0.035162	-	0.002583
hsa05014	Amyotrophic lateral sclerosis (ALS)	-	0.002728	-	-
hsa04210	Apoptosis	-	0.000471	0.012766	0.012915
hsa04976	Bile secretion	-	0.004412	-	0.020384
hsa04022	cGMP-PKG signaling pathway	-	-	0.012766	-
hsa05204	Chemical carcinogenesis	-	0.005466	0.000060	0.000001
hsa04625	C-type lectin receptor signaling pathway	-	0.010189	0.004639	0.002583
hsa00982	Drug metabolism - cytochrome P450	-	0.023850	0.000358	0.000095
hsa04915	Estrogen signaling pathway	-	-	0.000003	0.000166
hsa00071	Fatty acid degradation	0.002294	0.000044	0.000358	0.000033
hsa04540	Gap junction	-	-	-	0.006371
hsa00010	Glycolysis / Gluconeogenesis	-	-	0.031676	0.003194
hsa05167	Kaposi sarcoma-associated herpesvirus infection	-	-	-	0.001593
hsa04720	Long-term potentiation	-	-	0.001408	0.012915
hsa00980	Metabolism of xenobiotics by cytochrome P450	-	0.004412	0.000043	0.000001
hsa04928	Parathyroid hormone synthesis, secretion and action	0.002294	0.022929	0.000358	0.040149
hsa04070	Phosphatidylinositol signaling system	0.002294	0.002728	0.009600	-
hsa00830	Retinol metabolism	0.002294	0.000054	0.000003	0.000000
hsa05150	Staphylococcus aureus infection	-	-	0.003202	0.015063
hsa05216	Thyroid cancer	-	-	0.003116	-
hsa04918	Thyroid hormone synthesis	-	0.001620	0.001895	-
hsa00350	Tyrosine metabolism	0.003459	0.004412	0.005169	0.000052
hsa05135	Yersinia infection	-	0.002728	-	-
