# Supplementary material for: Genomic analyses of 10,376 individuals in the Westlake BioBank for Chinese (WBBC) pilot project
Source: Nat Commun. 2022 May 26;13:2939. doi: 10.1038/s41467-022-30526-x (PMC9135724; doi:10.1038/s41467-022-30526-x)
Supplement: Supplementary file 3 — Description of Additional Supplementary Files [file 41467_2022_30526_MOESM3_ESM.pdf]

Description of Additional Supplementary Files

File Name: Supplementary Data 1
Description: Summary of autosomal variants in each individual in the WBBC cohort

File Name: Supplementary Data 2
Description: The pathogenic and likely pathogenic variants were recorded by Clinvar in the 1,151 healthy individuals

File Name: Supplementary Data 3
Description: Top 1% of non-overlapping genomic windows was identified for positive selection in the North Han Chinese population using the iHS statistic. The adjacent regions, including the same genes, were merged. The clusters were sorted by the fraction of SNVs with |iHS| > 2.

File Name: Supplementary Data 4
Description: Top 1% of non-overlapping genomic windows was identified for positive selection in the Central Han Chinese population using the iHS statistic. The adjacent regions, including the same genes, were merged. The clusters were sorted by the fraction of SNVs with |iHS| > 2.

File Name: Supplementary Data 5
Description: Top 1% of non-overlapping genomic windows was identified for positive selection in the South Han Chinese population using the iHS statistic. The adjacent regions, including the same genes, were merged. The clusters were sorted by the fraction of SNVs with |iHS| > 2.

File Name: Supplementary Data 6
Description: Top 1% of non-overlapping genomic windows was identified for positive selection in the Lingnan Han Chinese population using the iHS statistic. The adjacent regions, including the same genes, were merged. The clusters were sorted by the fraction of SNVs with |iHS| > 2.
