# Supplementary material for: Genomic analyses of 10,376 individuals in the Westlake BioBank for Chinese (WBBC) pilot project
Source: Nat Commun. 2022 May 26;13:2939. doi: 10.1038/s41467-022-30526-x (PMC9135724; doi:10.1038/s41467-022-30526-x)
Supplement: Supplementary file 10 — Reporting Summary [file 41467_2022_30526_MOESM10_ESM.pdf]

Corresponding author(s): Hou-Feng Zheng

Last updated by author(s): Apr 20, 2022

Reporting Summary

Nature Portfolio wishes to improve the reproducibility of the work that we publish. This form provides structure for consistency and transparency in reporting. For further information on Nature Portfolio policies, see our Editorial Policies and the Editorial Policy Checklist.

Statistics

For all statistical analyses, confirm that the following items are present in the figure legend, table legend, main text, or Methods section.

n/a	Confirmed	
☐	☒	The exact sample size (*n*) for each experimental group/condition, given as a discrete number and unit of measurement
☐	☒	A statement on whether measurements were taken from distinct samples or whether the same sample was measured repeatedly
☐	☒	The statistical test(s) used AND whether they are one- or two-sided<br>*Only common tests should be described solely by name; describe more complex techniques in the Methods section.*
☒	☐	A description of all covariates tested
☐	☒	A description of any assumptions or corrections, such as tests of normality and adjustment for multiple comparisons
☐	☒	A full description of the statistical parameters including central tendency (e.g. means) or other basic estimates (e.g. regression coefficient) AND variation (e.g. standard deviation) or associated estimates of uncertainty (e.g. confidence intervals)
☐	☒	For null hypothesis testing, the test statistic (e.g. *F*, *t*, *r*) with confidence intervals, effect sizes, degrees of freedom and *P* value noted<br>*Give P values as exact values whenever suitable.*
☐	☒	For Bayesian analysis, information on the choice of priors and Markov chain Monte Carlo settings
☐	☒	For hierarchical and complex designs, identification of the appropriate level for tests and full reporting of outcomes
☒	☐	Estimates of effect sizes (e.g. Cohen's *d*, Pearson's *r*), indicating how they were calculated

Our web collection on statistics for biologists contains articles on many of the points above.

Software and code

Policy information about availability of computer code

Data collection	no software was used
Data analysis	ADMIXTURE v.1.3.0 (Alexander et al., 2009)<br>ANNOVAR 2019Oct24 (Wang et al., 2010)<br>BCFtools v.1.7 (Li et al., 2009)<br>BEAGLE v.5.1 (Browning et al., 2018)<br>BWA v.0.7.17 (Li and Durbin, 2010)<br>ClusterProfiler v.3.16.0 (Yu et al., 2012)<br>EIGENSOFT v.6.1.4 (Price et al., 2006)<br>GATK v.4.1.4.0 (Van der Auwera et al., 2013)<br>GCTA v.1.91 (Yang et al., 2011)<br>KING v.2.2.4 (Manichaikul et al., 2010)<br>Minimac3 v.2.0.1 (Das et al., 2016)<br>Minimac4 v.1.0.2 (Das et al., 2016)<br>Plink v.1.9 (Chang et al., 2015)<br>R v.4.0.2 package "mathii/saltice" (Mathieson et al., 2013)<br>Refined IBD v.17Jan20.102 (Browning and Browning, 2013)<br>REHH v.3.1.0 (Gautier et al., 2017)<br>SAMtools v.1.7 (Li et al., 2009)<br>SDS v.Sep.19.2016 (Field et al., 2016)<br>SHAPEIT v.2r900 (Delaneau et al., 2013)<br>SMC++ v.1.15.2 (Terhorst et al., 2017)

TreeMix v.1.13 (Pickrell and Pritchard, 2012)
VCFtools v.0.1.13 (Danecek et al., 2011)
VerifyBamID v 1.1.3 (Jun et al., 2012)
The scripts for R (version 4.0.2) and variant calling pipeline used in this study can be found from our GitHub repository (https://github.com/peikuan/WBBC).

For manuscripts utilizing custom algorithms or software that are central to the research but not yet described in published literature, software must be made available to editors and reviewers. We strongly encourage code deposition in a community repository (e.g. GitHub). See the Nature Portfolio guidelines for submitting code & software for further information.

Data

Policy information about availability of data

All manuscripts must include a data availability statement. This statement should provide the following information, where applicable:

- Accession codes, unique identifiers, or web links for publicly available datasets
- A description of any restrictions on data availability
- For clinical datasets or third party data, please ensure that the statement adheres to our policy

The sequencing and vcf data generated in this study have been deposited in the Genome Sequence Archive in National Genomics Data Center China National Center for Bioinformation / Beijing Institute of Genomics, Chinese Academy of Sciences, under accession number HRA001385 (https://ngdc.cncb.ac.cn/gsa-human/). The Fastq data are available under restricted access for privacy protection and access can obtained by application on the website. The access authority can be obtained for Research Use Only. The processed frequency data are available at the WBBC website (https://wbbc.westlake.edu.cn/genotype.html). The online imputation service could also be available at the WBBC website (https://imputationserver.westlake.edu.cn/). Other data generated in this study are provided in the Supplementary Information/Source Data file. Source data are provided with this paper. The data used in this study are the reference genome GRCh38 (ftp://gsapubftp-anonymous@ftp.broadinstitute.org/bundle/), 1000 Genome Project (http://ftp.1000genomes.ebi.ac.uk/vol1/ftp/release/), gnomAD (http://www.gnomad-sg.org/downloads), UK10K (https://www.uk10k.org/data.html), CONVERGE Project (http://ftp.ebi.ac.uk/pub/databases/eva/PRJNA289433/), dbSNP Build 151 (https://hgdownload.soe.ucsc.edu/downloads.html) and Allen Ancient DNA Resource (https://reich.hms.harvard.edu/allen-ancient-dna-resource-aadr-downloadable-genotypes-present-day-and-ancient-dna-data, version 44.3).

Field-specific reporting

Please select the one below that is the best fit for your research. If you are not sure, read the appropriate sections before making your selection.

[x] Life sciences [ ] Behavioural & social sciences [ ] Ecological, evolutionary & environmental sciences

For a reference copy of the document with all sections, see nature.com/documents/nr-reporting-summary-flat.pdf

Life sciences study design

All studies must disclose on these points even when the disclosure is negative.

Sample size	We would like to recruit more diverse Han Chinese samples across China mainland. Both Westlake University and Xiangya Hospital contributed to the sample collection. We selected the samples from the WBBC pilot project across 29 of 34 administrative divisions in China (Provinces, Municipalities and Special Administrative Regions). The Xiangya Hospital contributed another 3,335 individuals from Hunan province.
Data exclusions	In our study, fifteen whole-genome sequencing individuals with FREEMIX score > 0.05 (contaminated samples) were excluded, and 40 duplicated samples or MZ twins were also excluded.
Replication	We replicated the population structure results using different methods. For judging the confidence in the analysis of genetic drift estimates between each province, ten bootstrap replicates were generated by setting the -bootstrap -k flag ranging from 10 to 100 (step-size = 10) to resample blocks of contiguous SNPs for drift parameter estimation.
Randomization	As we replied to the comments from reviewer 3, the imbalance distribution of the samples is a limitation in our study. In the 4334 Han Chinese individuals, 3074 individuals are from Hunan province, 703 are from Jiangxi province and 557 individuals are from other provinces. WBBC project will sequence more samples across the China mainland.
Blinding	In this study, the investigators tried to recruit more samples for covering the Han Chinese population in different provinces during data collection. And this study did not carry out the randomization trials on the participants.

Reporting for specific materials, systems and methods

We require information from authors about some types of materials, experimental systems and methods used in many studies. Here, indicate whether each material, system or method listed is relevant to your study. If you are not sure if a list item applies to your research, read the appropriate section before selecting a response.

Materials & experimental systems

n/a	Involved in the study
☒	☐ Antibodies
☒	☐ Eukaryotic cell lines
☒	☐ Palaeontology and archaeology
☒	☐ Animals and other organisms
☐	☒ Human research participants
☒	☐ Clinical data
☒	☐ Dual use research of concern

Methods

n/a	Involved in the study
☒	☐ ChIP-seq
☒	☐ Flow cytometry
☒	☐ MRI-based neuroimaging

Human research participants

Policy information about studies involving human research participants

Population characteristics	The WBBC pilot project of Westlake University has enrolled 14,726 individuals (4,751 males and 9,975 females aged 14-25 years) with diverse traits across 29 of 34 administrative divisions in China (Provinces, Municipalities and Special Administrative Regions). The Xiangya Hospital contributed another 3,335 individuals (1653 males and 1682 females aged 51-89 years) including 1,973 patients with Parkinson's disease and 1,362 health controls. These samples were included in WBBC later on. We now have added the information of age, sex and the diagnosis of the participants in the Supplementary Data 1.
Recruitment	The WBBC recruited more than ~14,000 individuals from the Jiangxi Medical College and Binzhou Medical University. The rest of samples, that is 3,335 individuals were recruited from the Xiangya Hospital. All the participants signed the consent forms before the sampling process. There is no potential self-selection bias or any other biases in our study. The details of the sample recruitment have been published elsewhere (PMID: 34183340).
Ethics oversight	The research program was approved by the Human Genetic Resource Administration of China (HGRAC: 2019-1962 and 2021-CJ1139), the Institutional Review Board of the Westlake University (2018-006) and Xiangya Hospital of Central South University (202005124).

Note that full information on the approval of the study protocol must also be provided in the manuscript.
